# A novel FBW7/NFAT1 axis regulates cancer immunity in sunitinib-resistant renal cancer by inducing PD-L1 expression

**DOI:** 10.1186/s13046-022-02253-0

**Published:** 2022-01-26

**Authors:** Wentao Liu, Dianyun Ren, Wei Xiong, Xin Jin, Liang Zhu

**Affiliations:** 1 Department of Urology, The Second Xiangya Hospital, Central South University, Hunan, Changsha, 410011 China; 2 Uro-Oncology Institute of Central South University, Changsha, 410011 Hunan China; 3 Department of pancreatic surgery, Union Hospital, Tongji Medical College, Huazhong University of Science and Technology, Wuhan, 430022 China

**Keywords:** NFAT1, Tyrosine kinase inhibitors, FBW7, PD-L1, RCC

## Abstract

**Background:**

Tyrosine kinase inhibitors (TKIs) alone and in combination with immune checkpoint inhibitors (ICIs) have been shown to be beneficial for the survival of metastatic renal cell carcinoma (mRCC) patients, but resistance to targeted therapy and ICIs is common in the clinic. Understanding the underlying mechanism is critical for further prolonging the survival of renal cancer patients. Nuclear factor of activated T cell 1 (NFAT1) is expressed in immune and nonimmune cells, and the dysregulation of NFAT1 contributes to the progression of various type of malignant tumors. However, the specific role of NFAT1 in RCC is elusive. As a regulator of the immune response, we would like to systemically study the role of NFAT1 in RCC.

**Methods:**

TCGA-KIRC dataset analysis, Western blot analysis and RT-qPCR analysis was used to determine the clinic-pathological characteristic of NFAT1 in RCC. CCK-8 assays, colony formation assays and xenograft assays were performed to examine the biological role of NFAT1 in renal cancer cells. RNA-seq analysis was used to examine the pathways changed after NFAT1 silencing. ChIP-qPCR, coimmunoprecipitation analysis, Western blot analysis and RT-qPCR analysis were applied to explore the mechanism by NAFT1 was regulated in the renal cancer cells.

**Results:**

In our study, we found that NFAT1 was abnormally overexpressed in RCC and that NFAT1 overexpression was associated with an unfavorable prognosis. Then, we showed that NFAT1 enhanced tumor growth and regulated the immune response by increasing PD-L1 expression in RCC. In addition, we demonstrated that NFAT1 was stabilized in sunitinib-resistant RCC via hyperactivation of the PI3K/AKT/GSK-3β signaling pathway. Furthermore, our study indicated that downregulation of the expression of FBW7, which promotes NFAT1 degradation, was induced by FOXA1 and SETD2 in sunitinib-resistant RCC. Finally, FBW7 was found to contribute to modulating the immune response in RCC.

**Conclusions:**

Our data reveal a novel role for the FBW7/NFAT1 axis in the RCC response to TKIs and ICIs. NFAT1 and its associated signaling pathway might be therapeutic targets for RCC treatment, especially when combined with ICIs and/or TKIs.

**Supplementary Information:**

The online version contains supplementary material available at 10.1186/s13046-022-02253-0.

## Background

Renal cell carcinoma (RCC) comprises 2.2% of all malignancies and is the ninth most common cancer worldwide [1]. The age at diagnosis is approximately 60, and twice as many men as women are diagnosed with RCC [2]. RCC is mainly divided into clear cell renal cell carcinoma (ccRCC) (∼75–80%) histological subtypes [3] and non-ccRCC histological subtypes, including papillary, chromophobe, collecting duct and unclassified subtypes [4]. Approximately 30% of patients with RCC present with metastatic disease, 25% present with locally advanced RCC and 45% present with localized disease [5]. The five-year survival rate of patients with metastatic renal cell carcinoma (mRCC) is between 0 and 20%, with a median overall survival of 10–15 months [6, 7].

Localized RCC can be successfully managed with surgery, whereas mRCC is refractory to conventional chemotherapy because of its intrinsic resistance to radiotherapy and chemotherapy. Limited systemic therapy options are available, and no chemotherapeutic regimen is accepted as a standard of care [8]. Hence, a better understanding of the detailed mechanisms underlying the pathogenesis of RCC is urgently required to identify more effective treatment strategies. Since hyperactivation of the vascular endothelial growth factor (VEGF) signaling pathway is a common feature of most RCCs due to biallelic *Von Hippel-Lindau* (*VHL*) gene defects, targeted therapies, such as VEGF monoclonal antibodies, tyrosine kinase inhibitors (TKIs) and mammalian target of rapamycin pathway inhibitors, have improved the treatment of RCC and reduced treatment toxicity over the past two decades [9]. Recently, immune checkpoint inhibitors (ICIs) alone and in combination with TKIs have been shown to be beneficial for the survival of mRCC patients and have been approved for first-line or second-line treatment of mRCC [10, 11]. However, primary or acquired resistance to targeted therapy and ICIs is common in the clinic [4]. Hence, understanding the underlying mechanism of target therapy resistance and the immune response in RCC is critical for further prolonging the survival of cancer patients.

The nuclear factor of activated T cell (NFAT) family was first described in T lymphocytes and contains five members, namely, NFAT1-NFAT5 [12]. NFAT1 (or NFATC2) was the first member of the NFAT family identified in T cells [12] and contains an N-terminal NFAT homology domain (NHD), a Rel homology domain (RHD) and a C-terminal domain [13]. The NHD domain of NFAT1 is bound by calcineurin to promote NFAT1 dephosphorylation and translocation to the nucleus, which regulates downstream gene expression upon calcium stimulation [13]. NFAT1 is expressed in immune and nonimmune cells and drives the regulation of innate and adaptive immune responses [14]. It is worth noting that dysregulation of NFAT1 contributes to the growth and invasion of glioma, breast cancer and melanoma [15–17]. Given that RCC has been demonstrated to be an immunogenic tumor, immune regulation is an important aspect of RCC initiation and development. However, the specific role of NFAT1, as a regulator of the immune response, in RCC is elusive.

In this study, we found that NFAT1 was abnormally overexpressed in RCC and that NFAT1 overexpression was associated with an unfavorable prognosis. Then, we showed that NFAT1 enhanced tumor growth and regulated the immune response in RCC. Moreover, we found that the protein expression of NFAT1 was upregulated in sunitinib-resistant RCC. Thus, we aimed to study the specific role of NFAT1 in TKIs resistance and ICIs response in RCC. Mechanically, we demonstrated that NFAT1 increased the PD-L1 expression level by initiating the transcription of *TNF*, and the dysregulated Phosphoinositide 3-kinase (PI3K)/AKT serine/threonine kinase (AKT)/ glycogen synthase kinase 3 beta (GSK-3β) signaling pathway in sunitinib-resistant RCC cells stabilizing NFAT1 via inhibiting the FBW7 function. Moreover, we showed that deregulation of NFAT1 might link TKI resistance and ICI resistance in RCC. Thus, NFAT1 and its associated signaling pathway might be therapeutic targets for RCC treatment.

## Materials and methods

### Cell culture

Two human renal cancer cell lines (786-O and ACHN) and a mouse renal cancer cell line (Renca) were periodically validated via short tandem repeat (STR) profiling (Procell, China). 786-O cells were cultured in RPMI-1640 medium (Gibco, USA) supplemented with 10% fetal bovine serum. ACHN cells were maintained in minimum essential medium (MEM) (Gibco, USA) with 10% fetal bovine serum. Renca cells were cultured in RPMI-1640 (Gibco, USA) supplemented with 10% fetal bovine serum (Gibco, USA), 0.1 mM NEAA, 1 mM sodium pyruvate, and 2 mM L-glutamine. All the cell lines were maintained at 37 °C and 5% CO_2_ in a humidified incubator.

### Chemicals and antibodies

MK 2206 (Cat# HY-108232), LY294002 (Cat# HY-10108), and a GSK-3β inhibitor (Cat# HY-13973A,) were purchased from MedChemExpress (USA). LiCl (Cat# L9650) was obtained from Sigma-Aldrich (USA), cycloheximide (CHX) (Cat# HY-12320) was purchased from MedChemExpress (USA), and MG132 (Cat# S2619) was obtained from Selleck (USA). A glyceraldehyde-3-phosphate dehydrogenase (GAPDH) antibody (Cat# 10494–1-AP, WB: 1:5000), an NFAT1 antibody (Cat# 24654–1-AP, IHC: 1:400, IP: 1:50), a PD-L1 antibody (Cat# 8076–1-AP, WB: 1:1000, IHC: 1:100), a AKT antibody (Cat# 10176–2-AP, WB: 1:1000), a phosphatase and tensin homolog (PTEN) antibody (Cat# 22034–1-AP, WB: 1:1000), an HA tag antibody (Cat# 51064–2-AP, WB: 1:1000), a F-box and WD repeat domain containing 7 (FBW7) antibody (Cat# 28424–1-AP, WB: 1:1000), an Enhancer of zeste 2 polycomb repressive complex 2 subunit (EZH2) antibody (Cat# 21800–1-AP, WB: 1:1000), a Myc tag antibody (Cat# 16286–1-AP, WB: 1:1000, IP: 1:50), a SET domain containing 2, histone lysine methyltransferase (SETD2) antibody (Cat# 55377–1-AP, WB: 1:1000), and a forkhead box A1 (FoxA1) antibody (Cat# 20411–1-AP, WB: 1:1000) were purchased from Proteintech. p-AKT S473 antibody (Cat# 4060, WB: 1:1000), GSK-3β antibody (Cat# 12456, WB: 1:1000, IP: 1:50) and human TNF (tumor necrosis factor)-α Neutralizing (D1B4) Rabbit antibody (Cat# #7321) were obtained from Cell Signaling Technology. A H3K36me3 antibody was purchased from Abcam (Cat# ab282572, WB: 1:1000).

### Quantitative real-time PCR (qRT-PCR)

Total RNA was extracted from cells and tissues using TRIzol reagent (Cat# 15596026, Invitrogen, USA) according to the manufacturer’s instructions. Reverse transcription and qRT-PCR were performed as previously described [18]. Reverse transcription was conducted to generate cDNA (PrimeScript™ RT reagent Kit). qRT-PCR analysis was carried out using TB Green™ Fast qPCR Mix. Relative mRNA levels of target genes were calculated using the 2-^ΔΔ^Cq method after normalization to GAPDH mRNA. The primer sequences used for qRT-PCR analysis are provided in Supplementary Table 1.

### Western blotting

Ethical approval for the use of human tissues (Renal cancer patients with or without sunitinib resistance) was obtained by the local ethics committee (The Second Xiangya hospital, China) (Approval No. 2021068). Written informed consent was acquired from all patients before surgery. We collected the specimens from patients with RCC that were diagnosed at the late stage and underwent a palliative resection of the tumor following sunitinib therapy. Postoperative imaging examination, such as computed tomography, was used to evaluate the therapeutic effect of sunitinib therapy. According to the response evaluation criteria in solid tumors (RECIST) version 1.1 [19], we defined those patients achieved complete remission or partial remission as sunitinib sensitive, and patients with progressive disease (PD) as sunitinib resistance.

Tumor tissues or cells lysates were obtained in RIPA buffer, freshly supplemented with 1 mM phenylmethanesulfonyl fluoride (PMSF). Protein concentration was assessed by the BCA method. Equal amounts of protein were separated by 10% sodium dodecyl sulfate-polyacrylamide gel electrophoresis (SDS-PAGE), transferred onto 0.45 μm polyvinylidene fluoride membranes (Millipore, USA), and incubated with the appropriate antibodies for more than 8 h at 4 °C. Next, membranes were probed with the appropriate secondary antibody for 1 h at room temperature. Protein signals were visualized using ECL detection reagent (Thermo Fisher Scientific, USA) and ChemiDoc XRS (Bio-Rad Laboratories, USA). GAPDH was used as a loading control. The results were analyzed with Image Lab Software.

### RNA interference for stable knockdown or overexpression target protein

Lentivirus-based small hairpin RNAs (shRNA) were from Sigma-Aldrich. psPAX2 and pMD2.G combined with specific shRNA were co-transfected into 293 T cells. Replace the culture medium with fresh DMEM with 10% FBS 24 h after transfection. 48 h later, the virus containing medium was collected and added to cancer cells with polybrene (12 μg/mL). Puromycin selection was performed at concentration ranges between 3 and 5 μg/mL 48 h after infection. The sequence information of shRNA is provided in Supplementary Table 2.

The pTsin lentiviral expression vector was used to generate lentiviral plasmids for Tsin-NFAT1, which could stably overexpress NFAT1 in cells as previously reported [20]. Lipofectamine 2000 was used to transfect 293 T cells with the pTsin expression plasmid and viral packaging plasmids (pHR’ CMVδ 9.8 and pVSV-G). Twenty-four hours after transfection, the medium was replaced with fresh DMEM, containing 10% FBS and 1 mM of sodium pyruvate. Next, 48 h post transfection, the virus culture medium was collected and added to renal cancer cells supplemented with 12 μg/ml of polybrene. Twenty-four hours after infection, the infected cells were selected with 10 μg/ml of puromycin.

### Syngeneic tumor model treatment protocol

All animal procedures were performed in a specific pathogen-free environment according to the guidelines of the Ethics Committee of Second Xiangya Hospital, Central South University (Changsha, China).

Athymic nude (nu/nu) mice (4–5 weeks old, male) were purchased from Vitalriver (Beijing, China). In total, 5 × 10^6^ 786-O cells were dispersed in 100 μL PBS and were subcutaneously injected into the left dorsal flank of nude mice. Tumor sizes were measured with a digital Vernier caliper at 2-day intervals. Tumor volumes were calculated using the following formula: tumor volume (mm3) = (L × W^2^) ÷ 2. Mice were sacrificed on day 21 or when tumor volume reached 1000 mm^3^.

Six-week-old C57BL/6 mice were purchased from Shulaobao Biotech (Wuhan, China). Renca cells (1 × 10^7^ in 100 μl 1 × PBS) infected with indicated lentivirus were injected s.c. into the right flank of mice. After the xenografts reached a size of approximately 50 mm^3^, mice carrying similar types of tumors were randomized into different groups and treated with anti-PD-1 (BioXcell, Clone RMP1–14)/IgG (BioXcell, Clone 2A3) (200 μg, i.p., given at days 0, 3, 6). Mice were euthanized and tumors were collected from all animals once the tumors reached a volume of 1000 mm^3^. The mass of the grafts was calculated from standard measurements.

### Statistical analysis

The replicants for each experiment were indicated in the figure legend. Parametric data are shown as the means ± standard deviation (SD), and nonparametric data are shown as the medians and ranges. Two-way ANOVA or one-way ANOVA with Tukey’s multiple comparison test was used for multiple group analysis. Unpaired Student’s *t*-tests were used to compare data between two groups. We considered the result to be statistically significant when *P* < 0.05. All statistical analyses were performed using GraphPad Prism 6 software (GraphPad Software, Inc., USA).

Other related methods were provided in the Supplementary information.

## Results

### Abnormal upregulation of NFAT1 expression enhances the proliferation of renal cancer cells

**Fig. 1:**
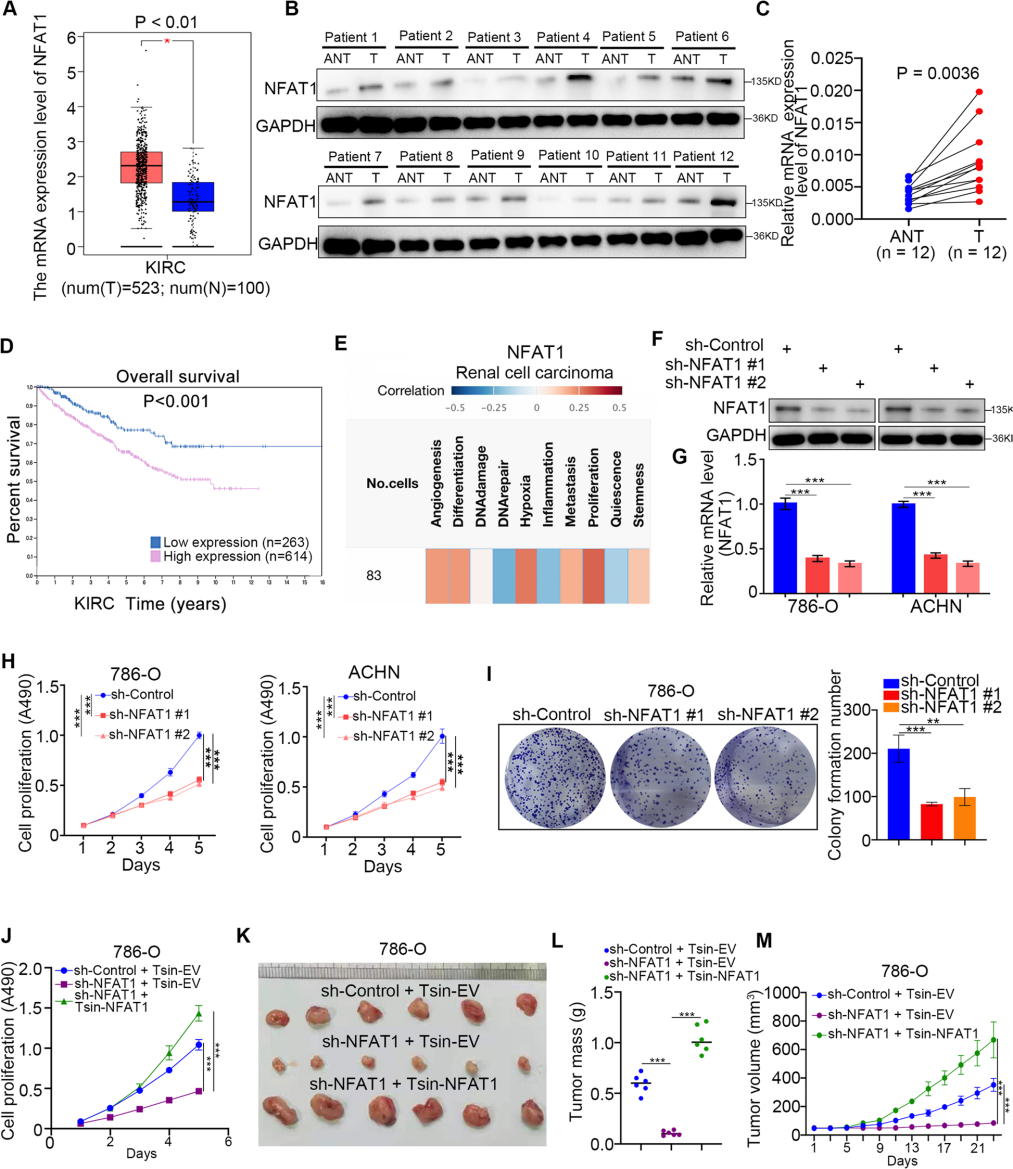
Abnormal upregulation of NFAT1 expression enhances the proliferation of renal cancer cells. **A** Determination of the NFAT1 mRNA expression level by the GEPIA web tool. Boxplot analysis of the expression level showing log2 (TPM + 1) on a log-scale. The NFAT1 expression was compared between ccRCC patient specimens and nontumor renal tissues. **B and C.** Western blot (**B**) and qRT-PCR analysis (**C**) of the protein and mRNA expression of NFAT1 in 12 paired renal cancer tissues (T) and the matched adjacent normal tissues (ANT) of the same patient. GAPDH served as an internal reference. ***, *P* < 0.001. The difference was compared between ANT group and tumor tissue group. **D** Determination of the overall survival NFAT1 mRNA expression level by the Human Protein Atlas database (P < 0.001) of RCC patients. The difference was compared between low NFAT1 expression and high NFAT1 expression group. **E** Determination of the biological role of NFAT1 by the CancerSea cancer single-cell state atlas (http://biocc.hrbmu.edu.cn/CancerSEA/) in RCC patients. **F and G.** Western blot (**F**) and qRT-PCR (**G**) analysis of NFAT1 expression in renal cancer cells infected with shControl or shNFAT1s. GAPDH served as an internal reference. For qRT-PCR analysis, data presented as the mean ± SD of three independent experiments. ***, *P* < 0.001. The differences were compared between shControl group and shNFAT1 group. **H and I.** Renal cancer cells infected with shControl or shNFAT1s were harvested for MTS (**H**) and colony formation assay assays (**I**). Each bar represents the mean ± SD of three independent experiments. **, *P* < 0.01 ***, P < 0.001. The differences were compared between shControl group and shNFAT1 group. **J** 786-O cells infected with or without shNFAT1 and/or Tsin-NFAT1 were harvested for MTS assay. ***, P < 0.001. The difference was compared between shControl+Tsin-EV and shNFAT1 + Tsin-EV group, or shNFAT1 + Tsin-EV and shNFAT1 + Tsin-NFAT1. **K-M.** 786-O cells infected with or without shNFAT1 and/or Tsin-NFAT1 were subcutaneously injected into nude mice. The tumors were harvested and photographed (**K**) on day 23. Data for tumor mass (**L**) and tumor volume (**M**) are shown as the mean ± SD (*n* = 6). ***, *P* < 0.001. The difference was compared between shControl+Tsin-EV and shNFAT1 + Tsin-EV group, or shNFAT1 + Tsin-EV and shNFAT1 + Tsin-NFAT1.

Currently, the cancer-related role of NFAT1 in ccRCC is unclear. We first performed bioinformatics analysis to evaluate the expression level of NFAT1 in cancer tissues and corresponding nontumor tissues by using the GEPIA web tool (http://gepia.cancer-pku.cn/) and TIMER web tool (http://cistrome.dfci.harvard.edu/TIMER/) (Fig. 1A and Supplementary Fig. 1A). Of note, we found that NFAT1 expression was abnormally upregulated in ccRCC patient specimens compared with nontumor renal tissues (Fig. 1A and Supplementary Fig. 1A). Similarly, we also showed that NFAT1 protein and expression levels were increased in ccRCC tissues compared with adjacent nontumor renal tissues collected at our hospital (Fig. 1B, C and Supplementary Fig. 1B). In addition, the Human Protein Atlas (https://www.proteinatlas.org/) dataset indicated that high expression levels of NFAT1 were associated with poor prognosis in patients with ccRCC (Fig. 1D). Thus, the above findings revealed that NFAT1 seems to be an oncogenic-like protein in RCC. Then, we aimed to study the biological role of NFAT1 in RCC cells. Intriguingly, the single-cell sequencing dataset from the cancer single-cell state atlas CancerSea (http://biocc.hrbmu.edu.cn/CancerSEA/) revealed that NFAT1 is involved in the regulation of angiogenesis, metastasis, and proliferation of multiple types of malignant tumors (Fig. 1E and Supplementary Fig. 1C-1E). We found that in RCC, NFAT1 is closely associated with the proliferation of cancer cells (Fig. 1E). To further evaluate the effect of NFAT1, we knocked down NFAT1 by infecting 786-O and ACHN cells with specific shRNAs (Fig. 1F and G). The MTS assay and colony formation assay demonstrated that knockdown of NFAT1 decreased the proliferation ability of RCC cells (Fig. 1H and I). Moreover, we rescued NFAT1 expression in 786-O cells by infecting them with a Tsin-NFAT1 construct and found that rescued of NFAT1 expression reversed the tumor-inhibiting effect of NFAT1 silencing in vitro and in vivo (Fig. 1J-M). Collectively, our data suggested that aberrantly expressed NFAT1 plays a role in modulating tumor growth in RCC.

### NFAT1 regulates the multiple signaling pathways associated with the immune response in RCC

**Fig. 2:**
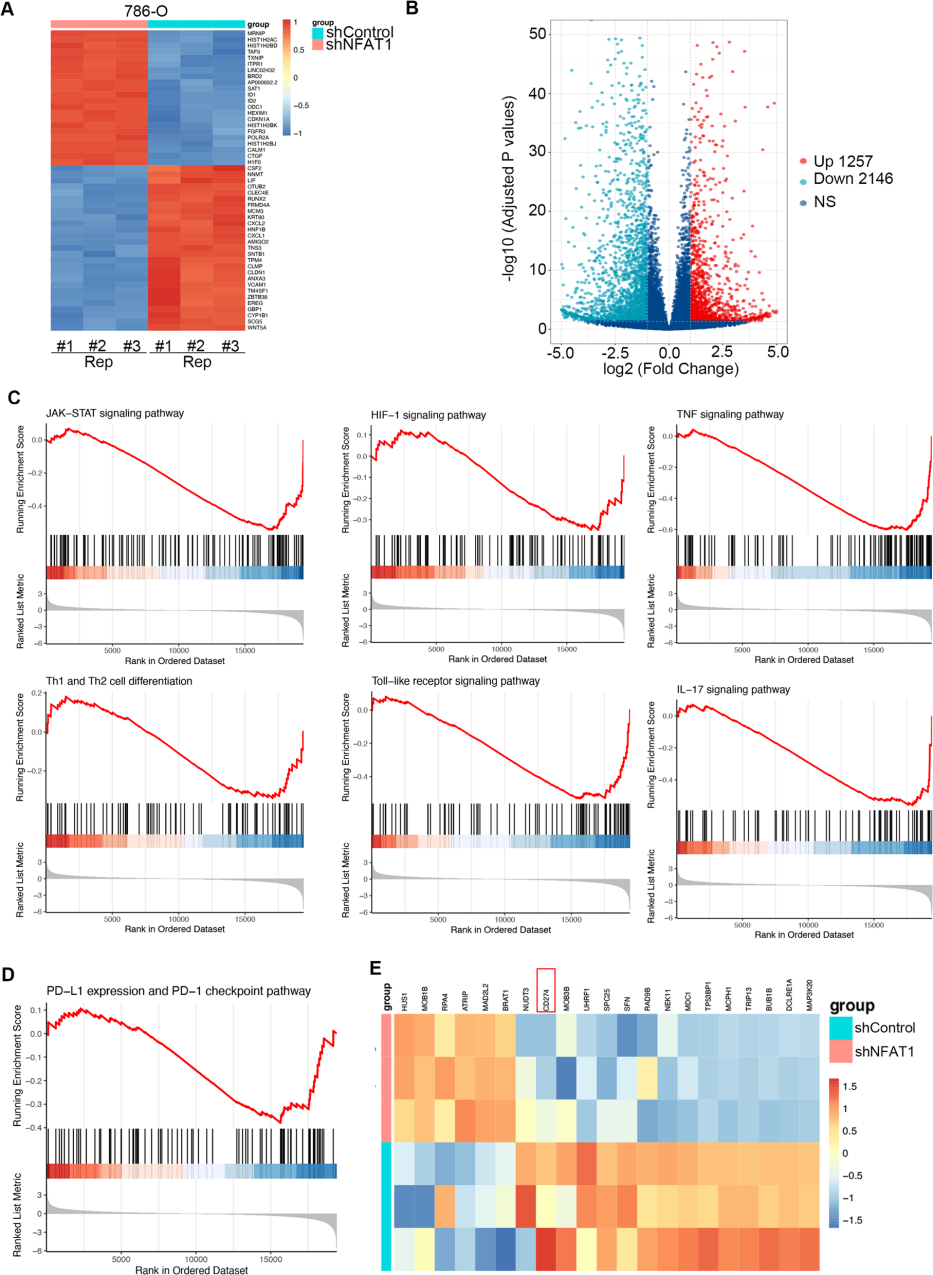
NFAT1 regulates the multiple signaling pathways associated with the immune response in RCC. **A and B** 786-O cells were infected with the indicated constructs for 72 h. Cells were subjected to RNA-seq analysis. **C** Gene Set Enrichment Analysis of JAK-STAT signaling pathway, HIF-1 signaling pathway, TNF signaling pathway, Th1 and Th2 cell differentiation, Toll-like signaling pathway, and IL-17 signaling pathway in NFAT1 silenced 786-O cells. **D** Gene Set Enrichment Analysis of PD-L1 expression and activation of PD-1 checkpoint pathway in NFAT1 silenced 786-O cells. **E** Heatmap to show the differential expressed genes of PD-L1 expression and activation of PD-1 checkpoint pathway in NFAT1 silenced 786-O cells

Given that NFAT1 acts as a growth-promoting protein in RCC, we aimed to uncover the underlying mechanism. First, RNA sequencing (RNA-seq) of 786-O cells was performed after NFAT1 knockdown. The genes that showed upregulated and downregulated expression after NFAT1 silencing are indicated in Fig. 2A and B. Then, we showed that knockdown of NFAT1 was associated with various inflammatory and immune response-related pathways, including the JAK-STAT signaling pathway, the HIF-1 signaling pathway, the TNF signaling pathway, Th1 and Th2 cell differentiation, the Toll-like signaling pathway and the IL-17 signaling pathway, by using Gene Set Enrichment Analysis (GSEA) (Fig. 2C). Since immune checkpoint therapy significantly improves the survival time of RCC patients [21, 22], we were curious about whether there is a relationship between NFAT1 and the PD-1 checkpoint pathway. Not surprisingly, NFAT1 was found to be involved in upregulating PD-L1 expression and activating the PD-1 checkpoint pathway (Fig. 2D and E). Thus, our data demonstrated that NFAT1 has a close relationship with various types of immune response pathways in RCC cells.

### NFAT1 increases PD-L1 expression via upregulation of TNF expression in RCC cells

**Fig. 3:**
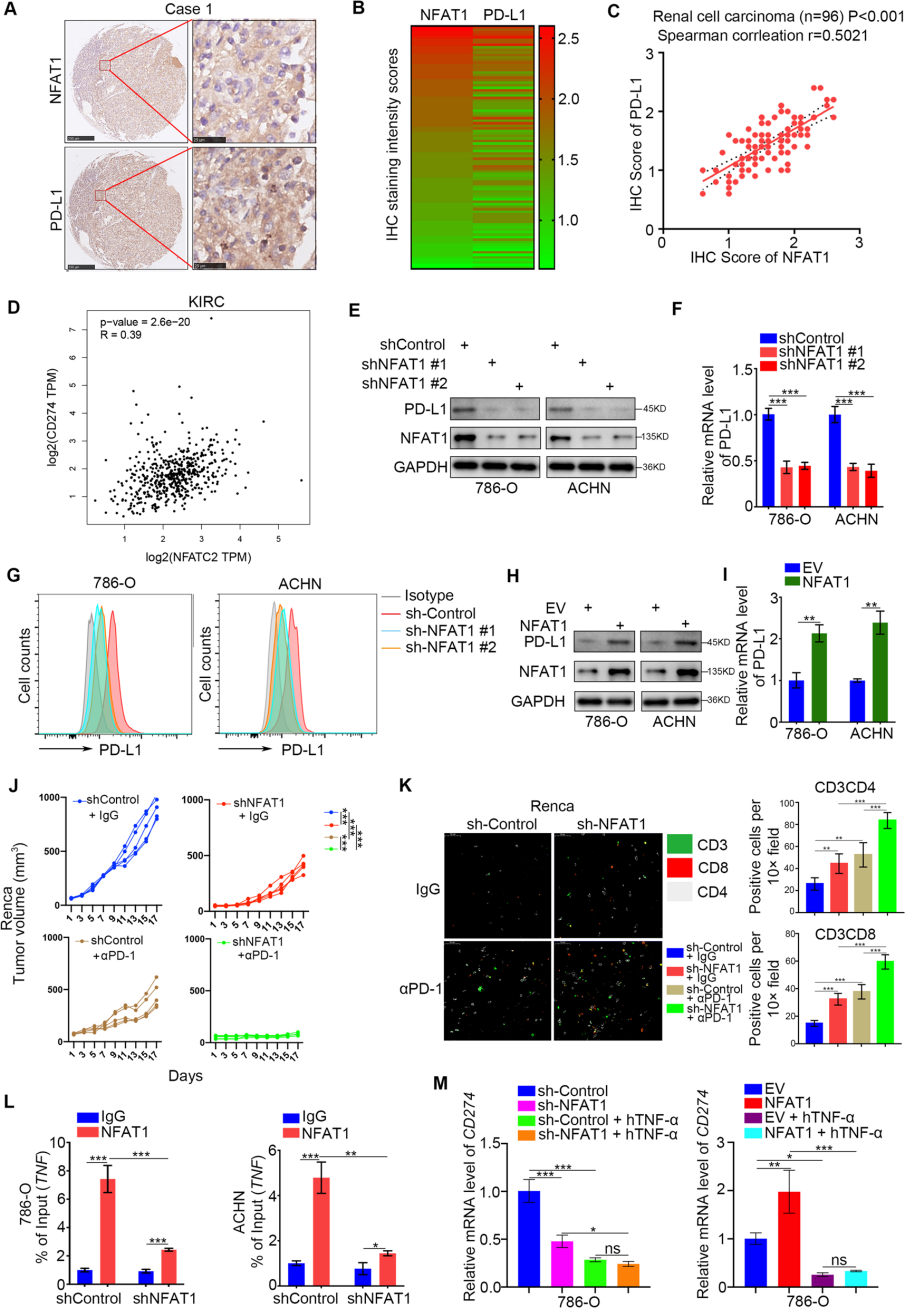
NFAT1 increases PD-L1 expression via upregulation of TNF expression in RCC cells. **A** IHC Images of NFAT1 and PD-L1 staining using TMA tissue sections (*n* = 96 RCC). The scale bars were shown as indicated. **B and C.** Heatmap (**B**) and dot plot (**C**) to show the correlation of IHC scores for the expression of the PD-L1 and NFAT1 proteins in RCC patient specimens. (*r* = 0.5021 for spearman correlation coefficients, *P* < 0.001). **D** The GEPIA web tool was searched for the correlation between the expression of PD-L1 and NFAT1 in mRNA levels in RCC samples. *P* values as indicated in the Fig. **E-G.** Western blot (**E**), qRT-PCR (**F**) and FACS analysis (**G**) of PD-L1 expression in renal cancer cells infected with shControl or shNFAT1s. GAPDH served as an internal reference. For qRT-PCR analysis, data presented as the mean ± SD of three independent experiments. ***, *P* < 0.001. **H and I.** Western blot (**H**) and qRT-PCR (**I**) analysis of PD-L1 expression in renal cancer cells infected with EV or NFAT1 plasmids. GAPDH served as an internal reference. For qRT-PCR analysis, data presented as the mean ± SD of three independent experiments. ***, *P* < 0.001. The differences were compared between shControl group and shNFAT1 group. **J** After 72 h of selection with puromycin, 5 × 10^6^ Renca cells infected with shControl or shNFAT1 were subcutaneously injected into the right dorsal flank of C57BL/6 mice. Mice with subcutaneous Renca tumors (*n* = 5/group) were treated with anti-PD-1 (200 μg) or nonspecific IgG for three times at day 2, 4, and 7. The mean of each group was compared with the mean of every other group. **K** Immunofluorescence staining analysis of the percentage of CD3^+^CD4^+^ and CD3^+^CD8^+^ T cells infiltrated in Renca tumors. Data are presented as the mean ± SD of five independent experiments (***, P < 0.001). **L** ChIP-qPCR of TNF in 786-O and ACHN cells. All data are shown as the mean values ± SD from three replicates. ns not significant; ***p < 0.001, unpaired t test. The difference was compared between IgG group and NFAT1 group, or shControl group and shNFAT1 group. **M** 786-O cells were infected or transfected with indicated constructs. Then these cells were treated with or without TNF-α neutralized antibody (50 pg/ml) for 24 h. qRT-PCR analysis of PD-L1 expression in 786-O and ACHN cells. GAPDH served as an internal reference. Data presented as the mean ± SD of three independent experiments. ***, *P* < 0.001. The mean of each group was compared with the mean of every other group.

We further explored the relationship between NFAT1 and PD-L1 in RCC. We first measured the protein levels of NFAT1 and PD-L1 in an RCC tissue microarray by IHC (Fig. 3A and B). Our results indicated that NFAT1 expression was positively correlated with PD-L1 expression (Spearman correlation *r* = 0.5021, *P* < 0.001, *n* = 96) (Fig. 3C). In addition, the GEPIA web tool analysis showed that the mRNA levels of NFAT1 (NFATC2) were positively correlated with the expression of PD-L1 (CD274) in ccRCC, lung adenocarcinoma, bladder cancer, liver cancer, prostate cancer, pancreatic cancer, and breast cancer (Fig. 3D, Supplementary Fig. 2A-2F). Then, we found that knockdown of NFAT1 by shRNAs decreased PD-L1 expression in both 786-O and ACHN cells (Fig. 3E-G). In contrast, overexpression of NFAT1 resulted in upregulation of PD-L1 expression in RCC cells (Fig. 3H and I). Besides, the overexpression of NFAT1 reversed the PD-L1 expression inhibition induced by NFAT1 silencing in RCC cells (Supplementary Fig. 2G-2H), as well as the main downstream molecules of PD-1 checkpoint pathway, *DCLRE1A* and *MAP 3 K20* (Supplementary Fig. 2I). Moreover, an in vivo experiment showed that NFAT1 silencing enhanced the antitumor effects of PD-1 antibodies and increased the CD3^+^CD4^+^ and CD3^+^CD8^+^ T lymphocytes infiltration in immune-competent mice (Fig. 3J and K). Thus, these data suggested that NFAT1 might participate in regulating PD-L1 expression in RCC. Since NFAT1 is known as a transcription factor, we next investigated whether NFAT1 directly initiates the transcription of PD-L1. Notably, ChIP-seq analysis of NFAT1 revealed no NFAT1 binding peak in the promoter region of PD-L1 (Supplementary Fig. 2 J). The above findings showed that NFAT1 has a close relationship with the JAK-STAT signaling pathway, HIF signaling pathway and TNF signaling pathway (Fig. 2C), which have been documented to regulate PD-L1 expression in cancer cells [23]. ChIP-seq analysis of NFAT1 showed that there were NFAT1 binding peaks on the promoters of *STAT3*, *HIF1A*, and *RELA* (Supplementary Fig. 3A). RNA-seq analysis of NFAT1 and a subsequent study demonstrated that knockdown of NFAT1 did not change the expression levels of STAT3, HIF1A, and RELA (Supplementary Fig. 3B). However, RNA-seq analysis of NFAT1 indicated that the expression of TNF, an activator of TNF signaling [24], was downregulated after NFAT1 silencing (Fig. 2). Moreover, there were NFAT1 binding peaks in the promoter region of *TNF* (Supplementary Fig. 3C). ChIP-qPCR showed that NFAT1 bound to the promoter region of *TNF* in both 786-O and ACHN cells (Fig. 3L). Knockdown of NFAT1 in 786-O and ACHN cells decreased TNF expression levels (Supplementary Fig. 3D). Furthermore, we showed that TNF-α neutralizing antibodies attenuated the decrease in PD-L1 expression induced by knockdown of NFAT1 in 786-O and ACHN cells (Fig. 3M). Together, these data showed that NFAT1 indirectly increases PD-L1 expression by activating the TNF pathway in RCC cells.

### NFAT1 is stabilized in sunitinib-resistant RCC cells via hyperactivation of the PI3K/AKT/GSK-3β signaling pathway

**Fig. 4:**
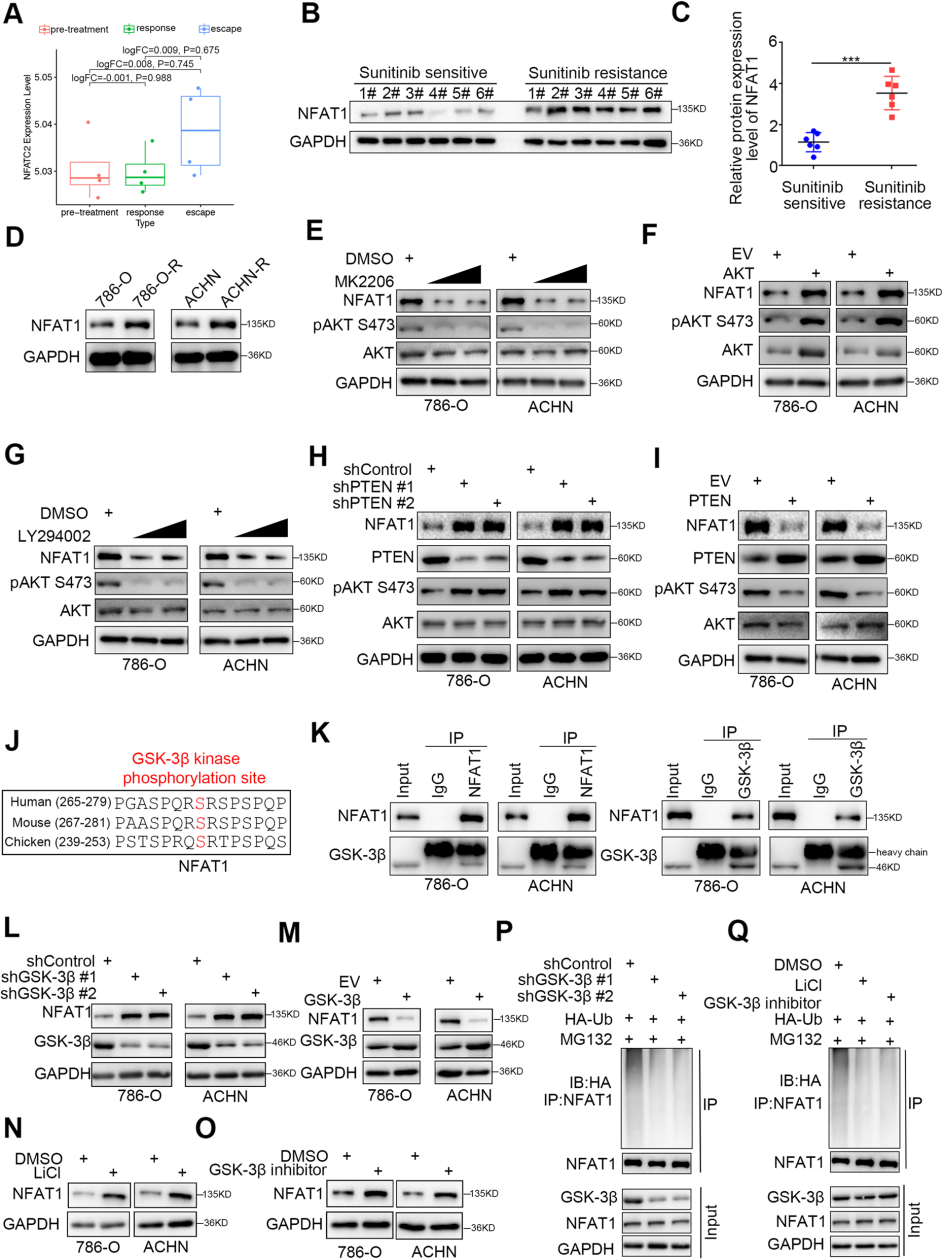
NFAT1 is stabilized in sunitinib-resistant RCC cells via hyperactivation of the PI3K/AKT/GSK-3β signaling pathway. **A** Relative mRNA expression level of NFAT1 in pre-treatment, response type and escape type RCC samples. The P values were shown as indicated. **B and C** Western blot analysis of NFAT1 expression in Sunitinib sensitive and resistance RCC patients. GAPDH served as an internal reference. ***, *P* < 0.001. The difference was compared between sunitinib sensitive group and sunitinib resistance group. **D** Western blot analysis of NFAT1 expression in Sunitinib sensitive and resistant 786-O and ACHN cells. GAPDH served as an internal reference. **E-I** Western blot analysis of NFAT1 expression in 786-O and ACHN cells. GAPDH served as an internal reference. **J** Amino acid sequence of NFAT1, in which found a consensus binding motif of GSK-3β. **K** co-immunoprecipitation assay to show the interaction between NFAT1 and GSK-3β. **L-O** Western blot analysis of NFAT1 expression in 786-O and ACHN cells. GAPDH served as an internal reference. **P and Q.** Western blot analysis in 786-O cells transfected with sh-GSK-3βs (**P**) or GSK-3β inhibitors (**Q**) for 48 h. Cells were treated with MG132 (10uM) for 8 h before harvested. GAPDH served as an internal reference

It has been reported that patients with mRCC benefit from PD-1 antibody treatment alone or in combination with TKIs [21, 22]. Thus, determining how to improve the antitumor effect of this combination therapy may further prolong the survival time of patients with mRCC. Various studies have reported that TKI resistance leads to an increase in PD-L1 expression [25], and upregulated PD-L1 expression in turn contributes to TKI resistance in cancer cells [26, 27]. Thus, we were curious about the regulatory network linking TKI resistance and PD-L1 expression. Here, we applied GSE76068 dataset to study the sunitinib-resistance associated genes, which analyzed the gene expression changes during development of sunitinib resistance in renal cell carcinoma patient derived xenografts (PDX). The GSE76068 dataset divide the PDX into three groups, namely pre-treatment phase (without Sunitinib treatment), response or sunitinib sensitive phase (treatment with Sunitinib 40 mg/kg/d p.o. and tumor exhibition a 91% reduction in volume), escape or sunitinib resistance phase (PDX in response phase treated with sunitinib for another 4 weeks and development resistance to sunitinib). We found that the mRNA expression level of NFAT1 was occasionally unchanged in PDX mouse models of sunitinib resistance phase and sunitinib sensitivity phase (Fig. 4A). We showed that the protein expression of NFAT1 was upregulated in sunitinib-resistant RCC specimens and cell lines (Fig. 4B-D). Dysregulation of the AKT signaling pathway is considered to be critically responsible for TKI resistance [28]. KEGG pathway enrichment analysis of the NFAT1 RNA-seq data showed that NFAT1 was associated with the AKT signaling pathway (Supplementary Fig. 4A and 4B). Interestingly, we found that knockdown of NFAT1 had no effect on the change in AKT phosphorylation but that ATK inhibitors downregulated the protein expression of NFAT1 in 786-O and ACHN cells (Supplementary Fig. 4C and Fig. 4E). In contrast, overexpression of AKT increased NFAT1 expression in RCC cells (Fig. 4F). Moreover, PI3K inhibitors decreased NFAT1 expression (Fig. 4G), and knockdown/overexpression of PTEN upregulated/downregulated NFAT1 expression in RCC cells (Fig. 4H, I). Given that the PI3K/AKT signaling axis has been reported to destabilize NFAT1 [29]**,** our data are consistent with previous findings. We further analyzed the amino acid sequence of NFAT1 and found that there was a consensus binding motif for GSK-3β, which is well known to be downstream of the PI3K/AKT signaling axis (Fig. 4J). Co-IP showed that NFAT1 interacts with GSK-3β in RCC cells (Fig. 4K). Knockdown of GSK-3β or treatment with a GSK-3β inhibitors increased NFAT1 protein levels, but overexpression of GSK-3β decreased NFAT1 expression in RCC cells (Fig. 4L-O). Furthermore, we demonstrated that GSK-3β silencing or GSK-3β inhibitor administration resulted in a decrease in NFAT1 ubiquitination in 786-O cells (Fig. 4P, Q). Taken together, our data suggested that NFAT1 is stabilized by the dysregulation of the PI3K/AKT/GSK-3β signaling pathway in RCC. However, it is not clear the transition from the in vitro model directly to the PDX Sunitinib resistant and not on anti-PD-1 treated mRCC patients.

### FBW7 promotes NFAT1 degradation in RCC cells

**Fig. 5:**
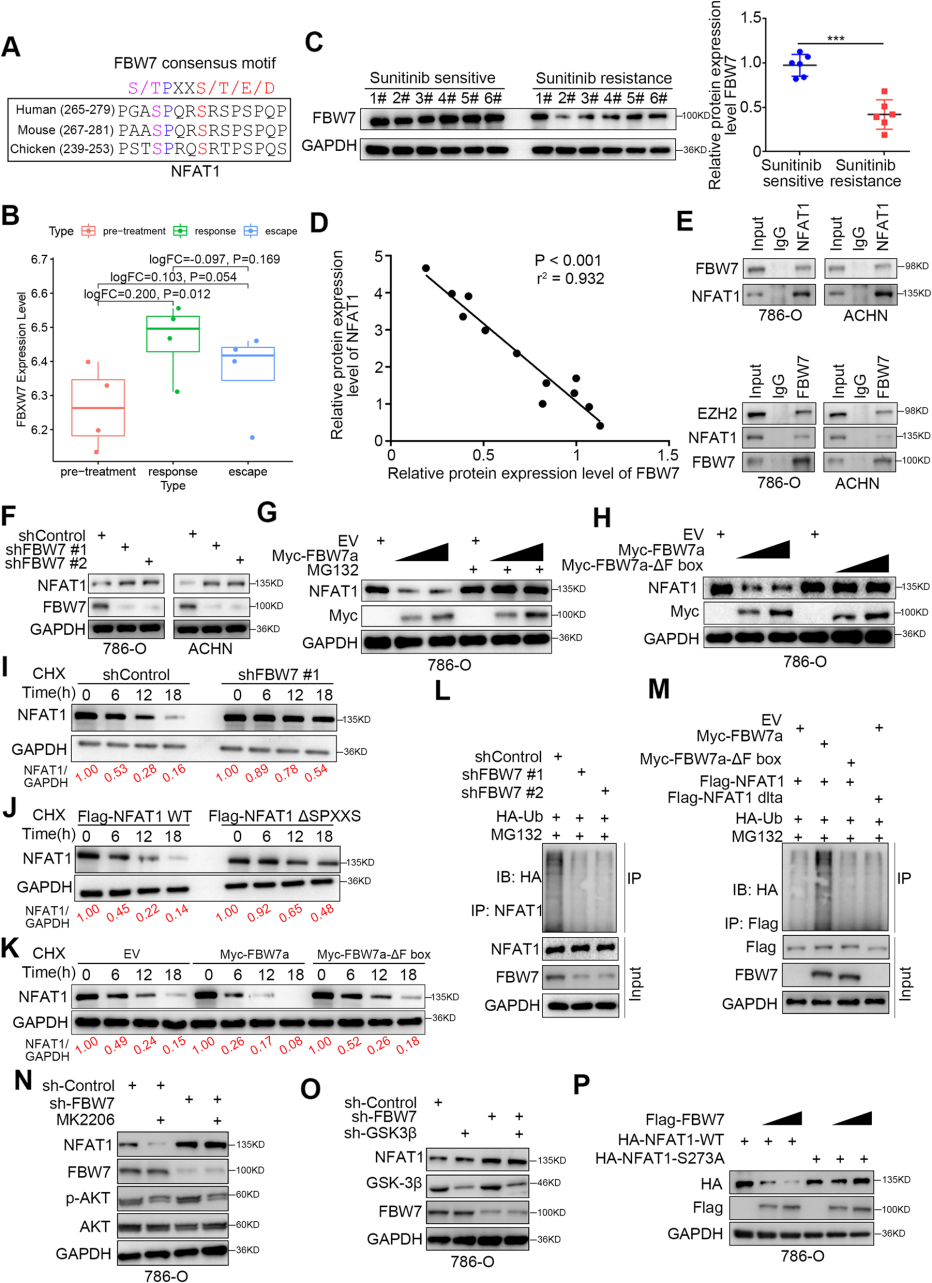
FBW7 promotes NFAT1 degradation in RCC cells. **A** Amino acid sequence of NFAT1, in which found a consensus binding motif of FBW7. **B** Relative mRNA expression level of FBW7 in pre-treatment, response type and escape type RCC samples. The P values were shown as indicated. The mean of each group was compared with the mean of every other group. **C** Western blot analysis of FBW7 expression in Sunitinib sensitive and resistance RCC patients. GAPDH served as an internal reference. ***, *P* < 0.001. The difference was compared between sunitinib sensitive group and sunitinib resistance group. **D** Scatter diagram to show the correlation between the protein expression level of NFAT1 and FBW7 in renal cancer patients. P values as indicated. **E** co-immunoprecipitation assay to show the interaction between NFAT1 and FBW7 in 786-O and ACHN cells. **F-K** Western blot analysis of NFAT1 expression in 786-O and ACHN cells with different treatment, which was indicated in the figure labels. GAPDH served as an internal reference. **L-M** Western blot analysis in 786-O cells transfected with shFBW7s (**L**) or FBW7 plasmids (**M**) for 48 h. Cells were treated with MG132 (10 uM) for 8 h before harvested. GAPDH served as an internal reference. **N** 786-O cells were infected with indicated constructs for 48 h. Then, cells were treated with or without MK2206 (10 uM) for another 24 h and subjected to Western blot analysis. **O** 786-O cells were infected with indicated constructs for 48 h. Cells were subjected to Western blot analysis. **P** 786-O cells were transfected with indicated constructs for 24 h. Cells were subjected to Western blot analysis

To explore the mechanism by which NFAT1 is degraded when the PI3K/AKT/GSK-3β signaling pathway is hyperactivated, we reanalyzed the amino acid sequence of NFAT1 and found that the FBW7 binding consensus motif *(T/S)PXX(S/T/D/E)* overlaps with the GSK-3β phosphorylation site mentioned above (Fig. 4 and 5J and A). Further analysis indicated that FBW7 expression was downregulated in the sunitinib-resistant mouse model and specimens from sunitinib-resistant RCC patients compared to the sunitinib-sensitive mouse model and specimens from sunitinib-resistant RCC patients (Fig. 5B and C). The protein levels of FBW7 were negatively correlated with those of NFAT1 in patients with RCC (Fig. 5D). FBW7 is a well-known E3 ligase that degrades multiple proteins, including Myc, JUN, Nothc1 and EZH2, in cancer cells [30]. We wondered whether NFAT1 is degraded by FBW7. First, co-IP showed that NFAT1 reciprocally interacts with FBW7 in RCC cells (Fig. 5E). Then, knockdown of FBW7 was found to increase NFAT1 protein levels in RCC cells (Fig. 5F). In contrast, overexpression of FBW7 reduced the protein levels of NFAT1, and this effect was diminished by proteasome inhibitor (MG132) treatment or transfection with the F-box-deleted FBW7 mutant (Fig. 5G and H). Furthermore, we showed that knockdown of FBW7 prolonged the protein half-life of NFAT1 in 786-O cells (Fig. 5I). In addition, deletion of the (SPXXS) binding sites in NFAT1 prevented a decrease in the protein level of NFAT1 (Fig. 5J). However, overexpression of wild-type (WT) FBW7 but not the F-box-deleted FBW7 mutant shortened the NFAT1 protein half-life in 786-O cells (Fig. 5K). Moreover, we showed that FBW7 silencing decreased the polyubiquitination of NFAT1 but that overexpression of FBW7 increased the polyubiquitination of NFAT1 in 786-O cells (Fig. 5L and M). Given that AKT/GSK-3β signaling pathway mediated the phosphorylation of S273 site of NFAT1. Consistent with the above finding, knockdown of FBW7 diminished the downregulation of NFAT1 induced by AKT inhibitors (MK2206, a highly selective inhibitors for AKT1/2/3) treatment or knockdown of GSK-3β in 786-O cells (Fig. 5N and O). Moreover, overexpression of FBW7 decreased the wild type NFAT1 but could not make effect on the S273A mutant of NFAT1, which mimic the de-phosphorylation status (Fig. 5P). Together, these data suggested that NFAT1 might be the substrate of FBW7 in RCC cells.

### FOXA1 and SETD2 induces downregulation of FBW7 expression in sunitinib-resistant RCC

We noted that FBW7 transcription was decreased in the RCC mouse model and tissues from sunitinib-resistant RCC patients (Fig. 5B and C). We next studied the underlying mechanism. Using existing ChIP-seq datasets in ChIP-Atlas, we found that a number of transcription factors and histones bind to the promoter region of *FBW7* (Supplementary Fig. 5A-B). The transcription factors and histones with the highest fold enrichment are indicated in Supplementary Fig. 5A. we first checked whether the expression of these genes (SETD2, FOXA1, ZIC2, ZFP42, SIX2, XRCC4) was changed between the sunitinib-resistant and sunitinib-sensitive groups. We found that only FOXA1 and SETD2 expression was decreased in the sunitinib-resistant group (Supplementary Fig. 5C-5 K). Of note, SETD2 trimethylates H3K36 (H3K36me3) to regulate FBW7 in mice [31] and FOXA1 recognizes H3K4me2 to regulate target gene expression [32]. Consistent with previous findings, knockdown of SETD2 decreased the FBW7 protein and mRNA levels in 786-O and ACHN cells (Supplementary Fig. 5 L and 5 M). Additionally, the GEPIA dataset indicated that SETD2 expression was positively correlated with FBW7 expression in ccRCC, prostate cancer, and bladder cancer (Supplementary Fig. 5 N). In addition, we showed that there were FOXA1 and H3K4me2 binding peaks in the promoter of FBW7, and subsequent ChIP-qPCR showed that FOXA1 binds to the promoter of FBW7 in RCC cells (Supplementary Fig. 6A-6C). Moreover, knockdown of FOXA1 decreased FBW7 expression in RCC cells (Supplementary Fig. 6D and 6E). Then, we also showed that FOXA1 was positively correlated with FBW7 expression in liver cancer, prostate cancer and bladder cancer by using the GEPIA web tool (Supplementary Fig. 6F). Thus, our results indicated that FOXA1 and SETD2 might be the transcription factors responsible for the downregulation of FBW7 expression in sunitinib-resistant RCC.

### FBW7 contributes to modulating the immune response in RCC

**Fig. 6:**
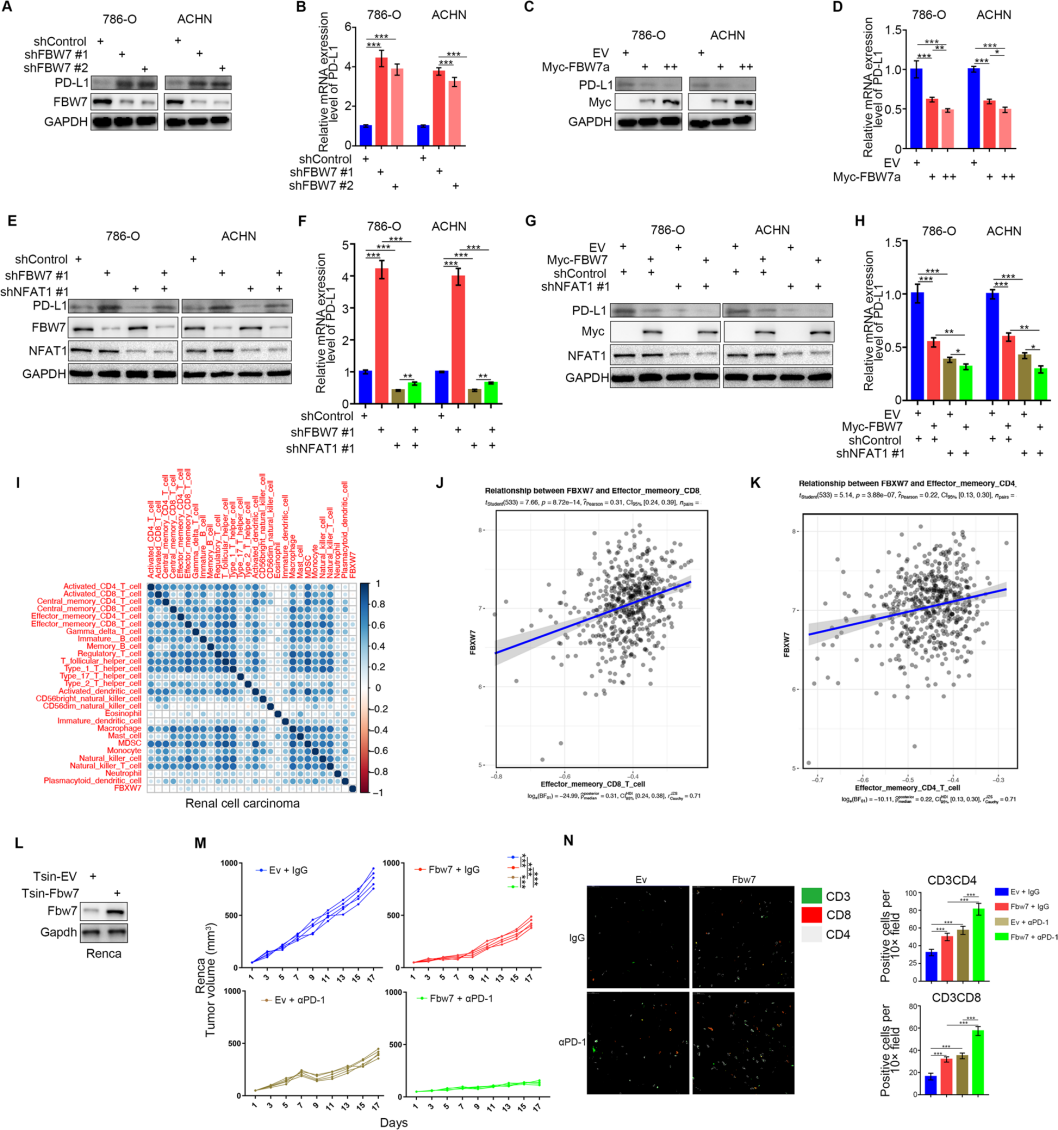
FBW7 contributes to modulating the immune response in RCC. **A and B.** Western blot (**A**) and qRT-PCR (**B**) analysis of PD-L1 expression in renal cancer cells infected with shControl or shFBW7s. GAPDH served as an internal reference. Data presented as the mean ± SD of three independent experiments. ***, *P* < 0.001. The difference was compared between shControl group and shFBW7 group. **C and D.** Western blot (**C**) and qRT-PCR (**D**) analysis of PD-L1 expression in renal cancer cells infected with EV or FBW7α plasmids. GAPDH served as an internal reference. Data presented as the mean ± SD of three independent experiments. ***, *P* < 0.001. The mean of each group was compared with the mean of every other group. **E and F.** Western blot (**E**) and qRT-PCR (**F**) analysis of PD-L1 expression in renal cancer cells infected with or without shFBW7 and/or shNFAT1 #1. GAPDH served as an internal reference. The mean of each group was compared with the mean of every other group. **G and H.** Western blot (**G**) and qRT-PCR (**H**) analysis of PD-L1 expression in renal cancer cells infected with or without FBW7 plasmids and/or shNFAT1 #1. GAPDH served as an internal reference. The mean of each group was compared with the mean of every other group. **I** The correlation between interested immune cell subsets and the expression of FBW7 in renal cell carcinoma. **J-K** TIMER database were searched to determine the correlation between CD4^+^ T cells (J) and CD8^+^ T cells (K) infiltration in RCC. **L** Western blot analysis of Fbw7 and PD-L1 expression in Renca cells infected with Ev or Fbw7 plasmids. GAPDH served as an internal reference. **M** After 72 h of selection with puromycin, 5 × 10^6^ Renca cells infected with Ev or Fbw7 plasmids were subcutaneously injected into the right dorsal flank of C57BL/6 mice. Mice with subcutaneous Renca tumors (*n* = 5/group) were treated with anti-PD-1 (200 μg) or nonspecific IgG for three times at day 1, 4, and 7. **N** Immunofluorescence staining analysis of the percentage of CD3^+^CD4^+^ and CD3^+^CD8^+^ T cells infiltrated in Renca tumors. Data are presented as the mean ± SD of five independent experiments (***, *P* < 0.001). The mean of each group was compared with the mean of every other group

Given that NFAT1 is closely associated with the immune response in RCC and is degraded by FBW7, we wondered whether FBW7 regulates the immune response in RCC. Not surprisingly, FBW7 silencing increased PD-L1 expression in 786-O and ACHN cells (Fig. 6A and B). In contrast, FBW7 overexpression resulted in downregulation of PD-L1 expression in RCC cells (Fig. 6C and D). Furthermore, we demonstrated that knockdown of NFAT1 partially attenuated the upregulation/downregulation of PD-L1 induced by knockdown/overexpression of FBW7 in 786-O and ACHN cells (Fig. 6E-H). Additionally, we showed that FBW7 expression was positively associated with CD4 and CD8 cell infiltration in RCC, liver cancer and pancreatic cancer (Fig. 6I-K, Supplementary Fig. 7A and 7B). Finally, mouse studies showed that overexpression of FBW7 enhanced the antitumor effect of PD-1 antibodies (Fig. 6L-M), and also further increased the CD3^+^CD4^+^ and CD3^+^CD8^+^ T lymphocytes infiltration induced by PD-1 antibodies in immune-competent mice (Fig. 6N). Taken together, these data indicated that FBW7 contributes to modulating the immune response in RCC partially through NFAT1.

## Discussion

Since a number of studies have demonstrated that RCC is unresponsive to chemotherapy, immunotherapy using interleukins or interferon was initially used to treat advanced RCC despite the poor outcomes [33, 34]. An improved understanding of genomic aberrations associated with RCC and the discovery of antiangiogenic molecules (such as TKIs and VEGFR monoclonal antibodies) have advanced the treatment of RCC, markedly improving survival [35]. RCC, especially ccRCC, is an immunogenic tumor with a large number of infiltrating immune cells, which makes ccRCC suitable for ICI therapy [36]. Recent clinical trials have shown that compared with the use of TKIs (sunitinib) alone, the use of PD-1 antibodies in combination with TKIs further improves the disease-free survival time of RCC patients [37]. This may be due to the interplay between the antigenic signaling pathway and immune-related pathway in cancer cells [38]. Resistance is also common after long-term use. Therefore, uncovering the associated mechanism is necessary for improving the antitumor effect of ICIs and TKIs. It has been documented that nonconding RNAs, RAF/MEK/ERK pathway, PI3K/AKT/mTOR pathway, hypoxic microenvironment are responsible for the TKIs resistance in RCC [39]. Modulation of PTEN/PI3K/AKT/mTOR signaling pathway could overcome the sunitinib resistance in renal cancer [39, 40]. Meanwhile, the dysregulation of PTEN/PI3K/AKT pathway is one of major cause for ICIs resistance in cancer [41, 42]. In consistence, we previously demonstrated that RRM2 could stabilize ANXA1 to activate PI3K/AKT signaling and lead to ICIs and sunitinib resistance in renal cancer [43]. Here, we found that NFAT1 expression was upregulated in sunitinib-resistant RCC due to hyperactivation of the PI3K/AKT pathway and downregulation of FBW7 expression. Abnormal overexpression of NFAT1 led to enhancement of PD-L1 expression, which is one of the mechanisms of ICI resistance in cancer, in RCC cells [44]. Furthermore, our result suggested that NFAT1 silencing significantly enhanced the antitumor effect αPD-1 in renal cancer xenografts. Thus, NFAT1 might play a certain role in modulating sensitivity to TKIs combined with ICIs in RCC.

It has been reported that NFAT1 is highly expressed and aberrantly activated in various human cancers, including breast cancer [45], pancreatic cancer [46], lung cancer [47], cervical cancer [48], and colon cancer [49], as well as melanoma [17], and contributes to the initiation, progression, and metastasis of these diseases. In addition, NFAT1 is dephosphorylated by Calcineurin and translocates to the nucleus, which it acts as a transcription factor and regulates the expression of many genes. Studies have reported that NFAT1 promotes melanoma metastasis by regulating autotaxin, IL-8 and MMP3 expression [17, 50]. Moreover, NFAT1 directly mediates IL-8 expression and secretion and increases neutrophil infiltration during breast cancer progression [16]. In addition, the DNA binding domain of NFAT1 can interact with Rel family proteins to initiate downstream gene expression. Notably, NFAT1 also resembles transcription factors of the AP-1 (Fos/Jun) family, allowing it to bind to certain regulatory elements of many immune response-related genes [51]. Interestingly, sorafenib treatment could activate NFAT1 and increase PD-1 expression in T cells in HCC [52]. Moreover, NFAT1 regulated the IL-6 signaling in glioma [53]. How does NFAT1 increase the expression of PD-L1 in RCC cells? Interestingly, we found that NFAT1 does not directly bind to the promoter of PD-L1, which indicates that PD-L1 is indirectly regulated by NFAT1. Furthermore, the KEGG pathway enrichment analysis of the NFAT1 RNA-seq dataset indicated that NFAT1 is involved in the regulation of the JAK-STAT, TNF and HIF signaling pathways, which have all been reported to regulate PD-L1 expression in cells [54–56]. A subsequent study showed that NFAT1 increases TNF transcription but not STAT3, HIF1a, or p65 transcription in RCC. Thus, our data provide relatively clear evidence for a regulatory axis between NFAT1 and PD-L1.

Given that NFAT1 is an ideal target for overcoming ICI resistance in RCC, elucidating the regulatory mechanism of NFAT1 has become crucial for identifying new therapeutic approaches. Li et al., reported that miR-155-5p/IRF2BP2 axis regulates the NFAT1 expression in cells [57]. Besides, Orai1 coupled with AKAP79 to regulate the activity of NFAT1 [58], and LMCD1 could dephosphorylate NFAT1 and promote NFTA1 nuclear translocation [59]. Here, we focused on the post-translational modification of NFAT1 in cells. We found that the PI3K/AKT/GSK-3β signaling axis regulated the stabilization of NFAT1 in sunitinib-resistant RCC cells, which is consistent with previous findings [29]. We further showed that the GSK-3β-induced destabilization of NFAT1 was mediated by FBW7. Since NFAT1 is a novel substrate of FBW7 and is closely associated with the immune response in RCC, the immune-related role of FBW7 attracted our attention. Intriguingly, subsequent analysis indicated that FBW7 expression was positively correlated with CD8 and CD4 T cell infiltration in renal cancer, liver cancer and pancreatic cancer. Additionally, we showed that FBW7 plays an important role in determining the PD-1 antibody response in renal cancer xenografts. Therefore, our data not only revealed one of the posttranscriptional modifications of NFAT1 but also provided a clue for understanding the immune-related role of FBW7 in RCC.

F-box and WD repeat domain-containing 7 (FBW7, also known as FBXW7), is a member of the F-box protein family, which constitutes one subunit of the Skp1, Cul1, and F-box protein (SCF) ubiquitin ligase complex [60, 61]. The role of FBW7 is to target the degradation of critical cellular regulators, thereby controlling essential cellular processes including cell cycle, DNA damage response, cell differentiation, apoptosis, and tumorigenesis [62, 63]. Since reduced FBW7 expression levels and loss-of-function mutations are found in a wide range of human cancers, FBW7 is generally considered as a tumor suppressor [62, 63]. In our study, we found that FBW7 was a novel E3 ligase for GSK-3β-induced degradation of NFAT1 in RCC cells, and FBW7 contributes to modulating the immune response in RCC to ICIs therapy partially through NFAT1. Besides, Fbw7 activity is controlled at different levels, resulting in specific and tunable regulation of the abundance and activity of its substrates [63]. Consistently, our result showed that downregulation of SETD2 and FOXA1 expression contributed to the decrease in FBW7 expression in sunitinib-resistant RCC cells, which indicated that the downregulation of FBW7 might be an important regulator of sunitinib-resistant in RCC cells. Therefore, FBW7 enhanced the response of RCC to TKIs and ICIs therapy and the treatment to upregulate FBW7 expression might represent a novel therapeutic strategy of sunitinib-resistant in RCC cells, especially combined with PD-1 antibody.

## Conclusion

**Fig. 7:**
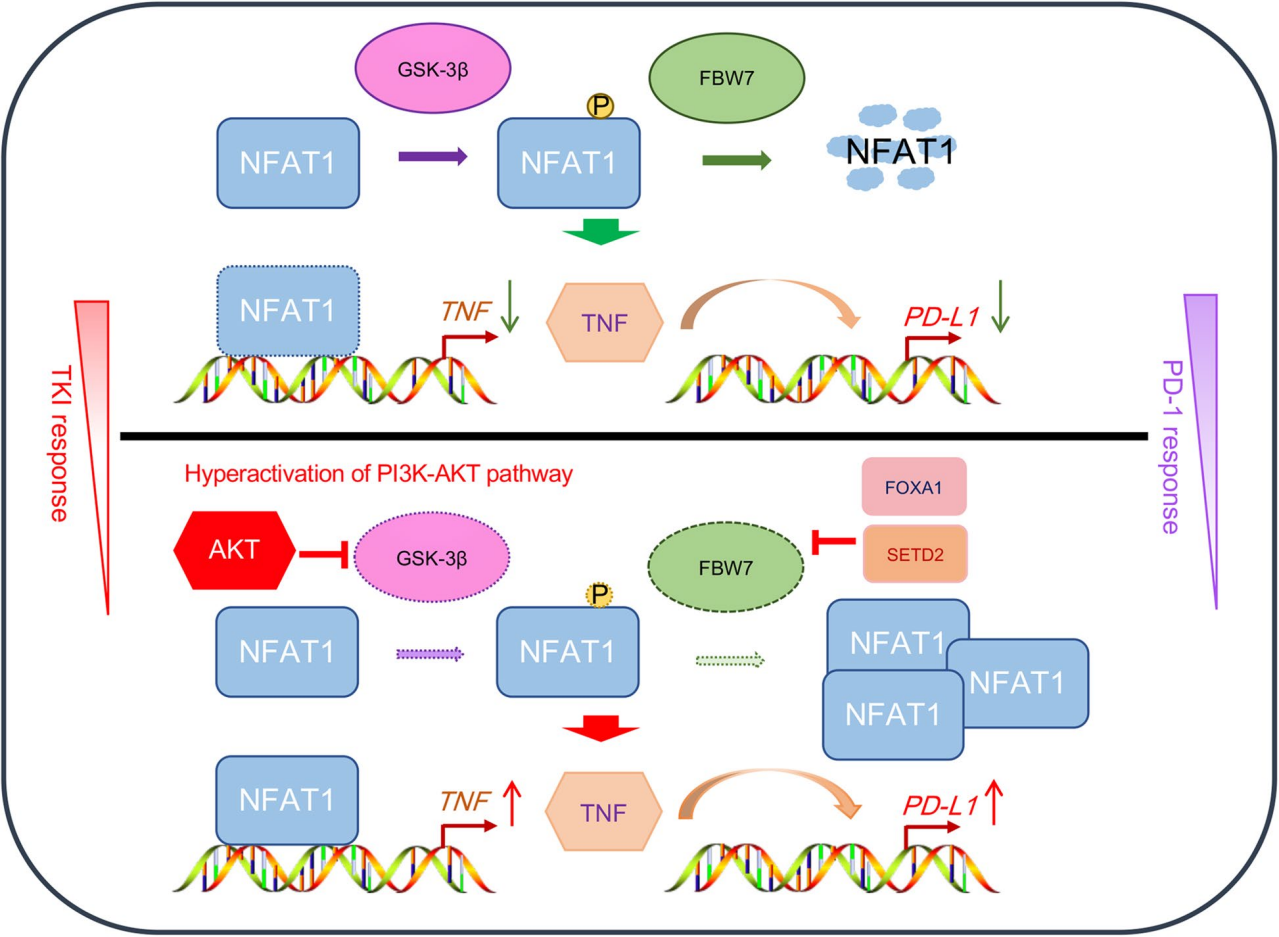
A model depicted that FBW7 was a novel E3 ligase for GSK-3β-induced degradation of NFAT1, and NFAT1 increased TNF expression to upregulate PD-L1 expression in RCC cells. Then, overactivation of the PI3K/AKT/GSK-3β signaling pathway stabilized NFAT1 in sunitinib-resistant RCC cells. Furthermore, we found that downregulation of SETD2 and FOXA1 expression contributed to the decrease in FBW7 expression in sunitinib-resistant RCC cells. Thus, the FBW7/NFAT1 axis played a role in the RCC response to TKIs and ICIs

Collectively, our results demonstrated that abnormal upregulation of NFAT1 expression is a predictor of an unfavorable prognosis in RCC and promotes the growth of renal cancer cells. We subsequently showed that NFAT1 increased TNF expression to upregulate PD-L1 expression in RCC cells. Then, we revealed that overactivation of the PI3K/AKT/GSK-3β signaling pathway stabilized NFAT1 in sunitinib-resistant RCC cells. Furthermore, we found that FBW7 was a novel E3 ligase for GSK-3β-induced degradation of NFAT1 in RCC cells. Moreover, we found that downregulation of SETD2 and FOXA1 expression contributed to the decrease in FBW7 expression in sunitinib-resistant RCC cells. We also showed that FBW7 was closely associated with immune cell infiltration and the PD-1 antibody response in renal cancer (Fig. 7). Thus, our data reveal a novel role for the FBW7/NFAT1 axis in the response of RCC to TKIs and ICIs therapy, and the treatment to target FBW7/NFAT1 axis might be a novel therapy strategy to enhanced the anti-tumor effect of PD-1 antibody, especially for sunitinib-resistant RCC patients.

## Supplementary Information

**Additional file 1.**

## Data Availability

Please contact the corresponding author (Xin Jin, jinxinxy2@csu.edu.cn) for data requests.

## Abbreviations

RCC: Renal cell carcinoma
ccRCC: Clear cell renal cell carcinoma
mRCC: Metastatic renal cell carcinoma
VEGF: Vascular endothelial growth factor
VHL: Von Hippel-Lindau
TKIs: Tyrosine kinase inhibitors
ICIs: Immune checkpoint inhibitors
NFAT: Nuclear factor of activated T cell
NHD: N-terminal NFAT homology domain
RHD: Rel homology domain
PI3K: Phosphoinositide 3-kinase
AKT: AKT serine/threonine kinase
GSK-3β: Glycogen synthase kinase 3 beta
STR: Short tandem repeat
MEM: Minimum essential medium
GAPDH: Glyceraldehyde-3-phosphate dehydrogenase
EZH2: Zeste 2 polycomb repressive complex 2 subunit
FBW7: F-box and WD repeat domain containing 7
SETD2: SET domain containing 2, histone lysine methyltransferase
FOXA1: Forkhead box A1
TNF: Tumor necrosis factor)
RECIST: Response evaluation criteria in solid tumors
PD: Progressive disease
PMSF: Phenylmethanesulfonyl fluoride
shRNA: Small hairpin RNAs
PDX: Patient derived xenografts
