# Supplementary material for: A novel FBW7/NFAT1 axis regulates cancer immunity in sunitinib-resistant renal cancer by inducing PD-L1 expression
Source: J Exp Clin Cancer Res. 2022 Jan 26;41:38. doi: 10.1186/s13046-022-02253-0 (PMC8790872; doi:10.1186/s13046-022-02253-0)
Supplement: Supplementary file 1 — Additional file 1. [file 13046_2022_2253_MOESM1_ESM.docx]

**Supplementary information**

**A novel FBW7/NFAT1 axis regulates cancer immunity in sunitinib-resistant renal cancer by inducing PD-L1 expression**

Wentao Liu, Dianyun Ren, Wei Xiong, Xin Jin, Liang Zhu

**Supplementary material and methods**

**Immunoprecipitation**

Cell lysates were prepared in radioimmunoprecipitation assay (RIPA) buffer (Cat# P0013C, Beyotime, China) supplemented with protease inhibitor cocktail (Cat# P1005, Beyotime, China) and Halt Phosphatase Inhibitor Cocktail (Cat# P1045, Beyotime, China). Primary antibody (0.5–2.0 µg; listed below) was added to the cell lysate, and the sample was incubated on ice overnight. The next day, 20–50 µL of Protein A beads (Cat# P2012, Beyotime, China) was added, and the sample was incubated under rotation at 4°C for 3 h. The next day, the beads were washed at least six times with lysate buffer on ice and then subjected to western blotting analysis.

**Flow cytometry**

786-O and ACHN cells infected with sh-Control or sh-NFAT1s were prepared and stained for [flow cytometry](https://www.sciencedirect.com/topics/biochemistry-genetics-and-molecular-biology/flow-cytometry) with the following antibodies: isotype APC anti-human IgG Fc Antibody (Biolegend, clone HP6017, USA) or APC anti-human PD-L1 antibody (Biolegend, clone 29E.2A3, USA) for 30 mins at room temperature. After washing with PBS three times, the cells were resuspended in 150 ul staining buffer and analyzed for flow cytometry. Events were acquired on a FACS Calibur flow cytometer (BD Biosciences) and analyzed with FlowJo software.

**Immunofluorescence**

Tumors removed from sacrificed mice were fixed for 3 days with 4% paraformaldehyde at 4℃, and then dehydrated before embedded in OCT compound. Cryosections were air-dried and used directly. The samples and cryostat microtome were prechilled to −20℃, and then tumor sections (45 µm) were cut on a cryostat microtome, blocked with 5% goat serums in PBS containing 0.1% Triton X-100 for 2 hours, and then incubated with primary antibodies (CD3, 1:200, CD4, 1:200; CD8, 1:100) in blocking buffer overnight. Subsequent to the excess primary antibody being washed off, sections were incubated with Alexa Fluor 647-labeled goat antirat IgG (Jackson ImmunoResearch, 1:200) and DAPI for 3 hours. The sections were sealed with antifluorescence quencher. After standing for a few days, fluorescence was visualized and images along the z-axis (z-axis interval: 2 µm) were captured by Olympus FV3000 inverted confocal microscope (Centre Valley, Pennsylvania, USA) equipped with a 40× objective lens. The cell number of positive signals per cubic millimeter was calculated.

**RNA sequencing**

A total of 1 µg of RNA per sample was used as the starting material for RNA sequencing (RNA-seq). RNA integrity was assessed using the RNA Nano 6000 Assay Kit of the Bioanalyzer 2100 system (Agilent Technologies, CA, USA). Clean data (clean reads) were obtained by removing reads containing adapter, reads containing ploy-N and low quality reads from raw data. At the same time, Q20, Q30 and GC content the clean data were calculated. All the downstream analyses were based on the clean data with high quality. Sequencing libraries were generated using the NEBNext Ultra RNA Library Prep Kit for Illumina (NEB, USA) following the manufacturer’s instructions, and index codes were added to attribute sequences to each sample. Clustering of the samples was performed on the cBot Cluster Generation System using the TruSeq PE Cluster Kit v3-cBot-HS (Illumina) according to the manufacturer’s instructions. After cluster generation, libraries were sequenced on an Illumina Novaseq platform, and 150-bp paired-end reads were generated. FeatureCounts v1.5.0-p3 was used to count the read numbers mapped to each gene. Differential expression analysis (two biological replicates per condition) was performed using the DESeq2 R package (1.16.1), and the cluster Profiler R package was used to test the statistical enrichment of differentially expressed genes (DEGs) in KEGG (Kyoto Encyclopedia of Genes and Genomes) pathways. Three replicates were performed in each group.

**Chromatin immunoprecipitation (ChIP) and ChIP-qRT-PCR**

ChIP assays were performed according to the instructions of the Pierce Magnetic ChIP Kit (Thermo Fisher Scientific, #26157). Cell samples (107 cells/sample) were crosslinked using 1% formaldehyde for 10 min. Glycine solution (10×) was added to each sample containing cell culture media and formaldehyde to a final concentration of 1×. The samples were mixed well and incubated at room temperature for 5 min. Diluted micrococcal nuclease was used to digest the DNA, and the nuclear membrane was broken using Scientz-ⅡD (10% power, 4 s on and 9 s off) for 2 min. After centrifugation, the supernatant was subjected to immunoprecipitation with anti-RNA polymerase II antibody, normal IgG, and primary antibodies at 4°C overnight. After IP elution and DNA recovery, the samples were collected for sequencing and real-time PCR analysis.

**Colony formation assay**

A total of 500 renal cancer cells were seeded in a 6-well cell culture cluster and cultured for 14 days. After 14 days, colonies were fixed with 4% paraformaldehyde for 30 min at room temperature, then stained with a phosphate-buffered saline (PBS) solution containing 0.1% crystal violet (Sigma-Aldrich) for 30 min. The samples were photographed, and visible colonies were counted.

**MTS assay**

The viability of cells seeded in 96-well plates was tested using 3-(4,5-dimethylthiazol-2-yl)-5-(3-carboxymethoxyphenyl)-2-(4-sulfophenyl)-2H-tetrazolium (MTS) reagent (Cat# ab197010, Abcam, USA). The renal cancer cells were incubated in 96-well plates with a density of 1×10^3^ cells/well and then allowed to grow for 0, 1, 2, 3, 4, and 5 days. MTS reagent (20 µL) was added to each well, and cells were incubated for 3 h. Optical absorbance at 490 nm was assessed on a microplate reader. All experiments were repeated three times.

**Immunohistochemistry (IHC)**

The tissue microarray slides were purchased from Avilabio (Kid-1921). IHC analysis was performed to determine the protein expression level with the NFAT1 and PD-L1 antibodies. Two independent pathologists, who were uninformed with the patient data and histopathological features of the samples, were responsible for reviewing and scoring the degree of immunostaining separately. Staining intensity was graded/scored in a blinded fashion: 1 = weak staining at ×100 magnification but little or no staining at ×40 magnification; 2 = medium staining at ×40 magnification; 3 = strong staining at ×40 magnification. A final staining index was calculated using the formula: staining intensity × percentage.

**Bioinformatic data mining**

The GEPIA web tool was used to determine the protein expression level in renal cancer. Human Protein Atlas cancer databases (https://www.proteinatlas.org/) were mined to predict the NFAT1 differential expression level between cancer and a healthy group. A UCSC Genome Browser was used to show the ChIP-seq signal profiles in the specific gene locus. Upstream targets of the key gene were determined by ChIP-seq and correlation analysis. Binding site in the targeted gene promoter of the key protein or methylation were obtained from ChIP-Atlas database (https://chip-atlas.org/). Correlation analysis between gene/protein and targeted genes was applied to further validate the ChIP-seq results. Bioinformatics analysis were carried out using the R Bioconductor (version 3.6.3). The gene expression data and clinical data of KIRC were downloaded through TCGA biolinks packages (version 2.14.1), and then the samples that did not meet the requirements were manually cleaned. The ssGSEA scores of immune cell subsets were calculated through the R GSVA package (version 1.34.0), and then the relationship between the ssGSEA scores of 27 immune cell subsets and the expression of FBXW7 were analyzed. The ggstatsplot package (version 0.6.5) were applied to plot the correlation between interested immune cell subsets and the expression of FBXW7.


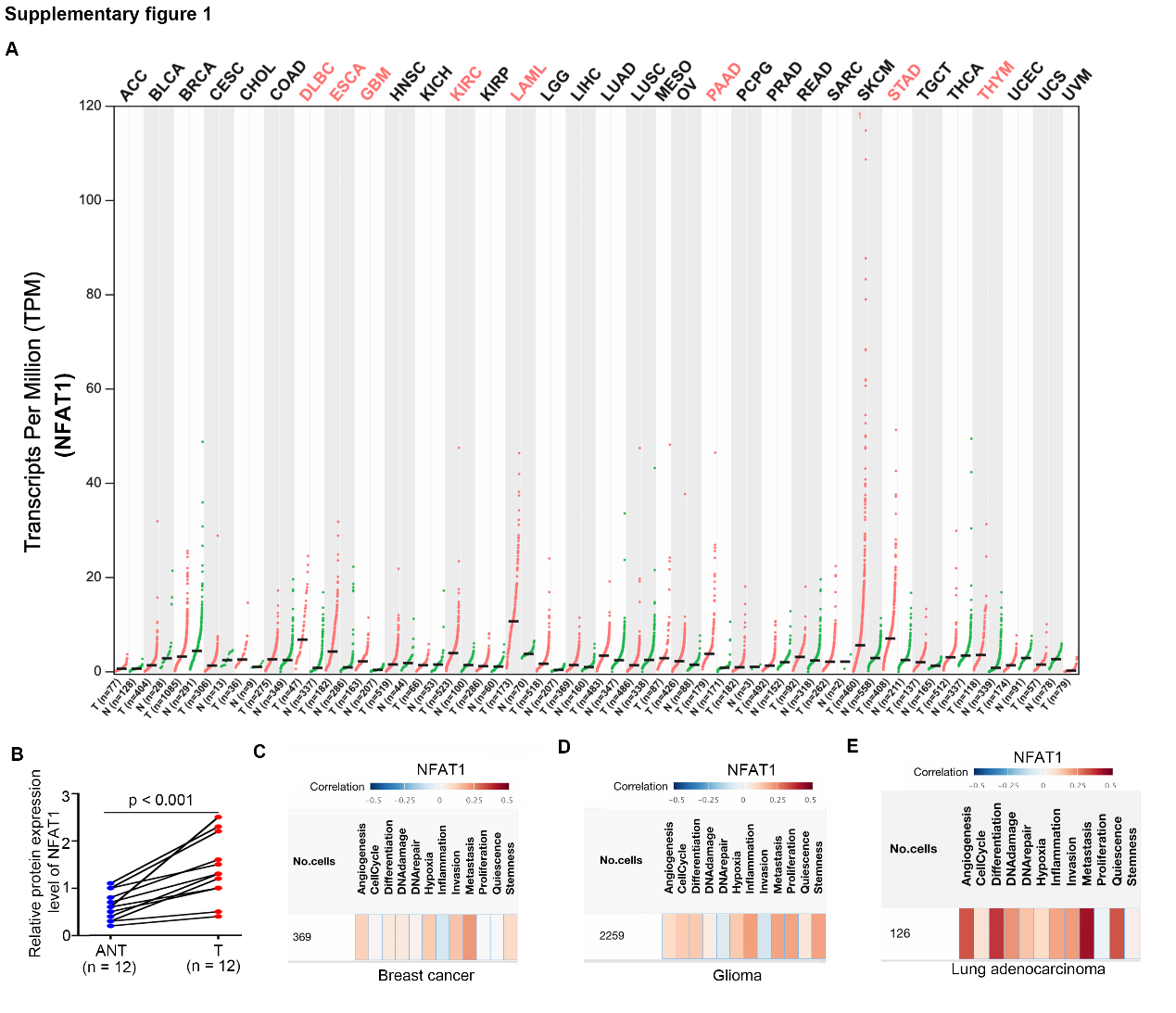
**Supplementary figure 1. The abnormally upregulated NFAT1 enhances the proliferation of renal cancer cells.**

**A.** Determination of the NFAT1 mRNA expression level in malignant cancers by the GEPIA web tool.

**B.** Relative protein expression level of NFAT1 of Figure 1B. The P value was indicated in the figure label.

**C-E.** Determination of the biological role of NFAT1 by the CancerSea cancer single-cell state atlas (http://biocc.hrbmu.edu.cn/CancerSEA/) in Breast cancer, Glioma, and lung adenocarcinoma patients.


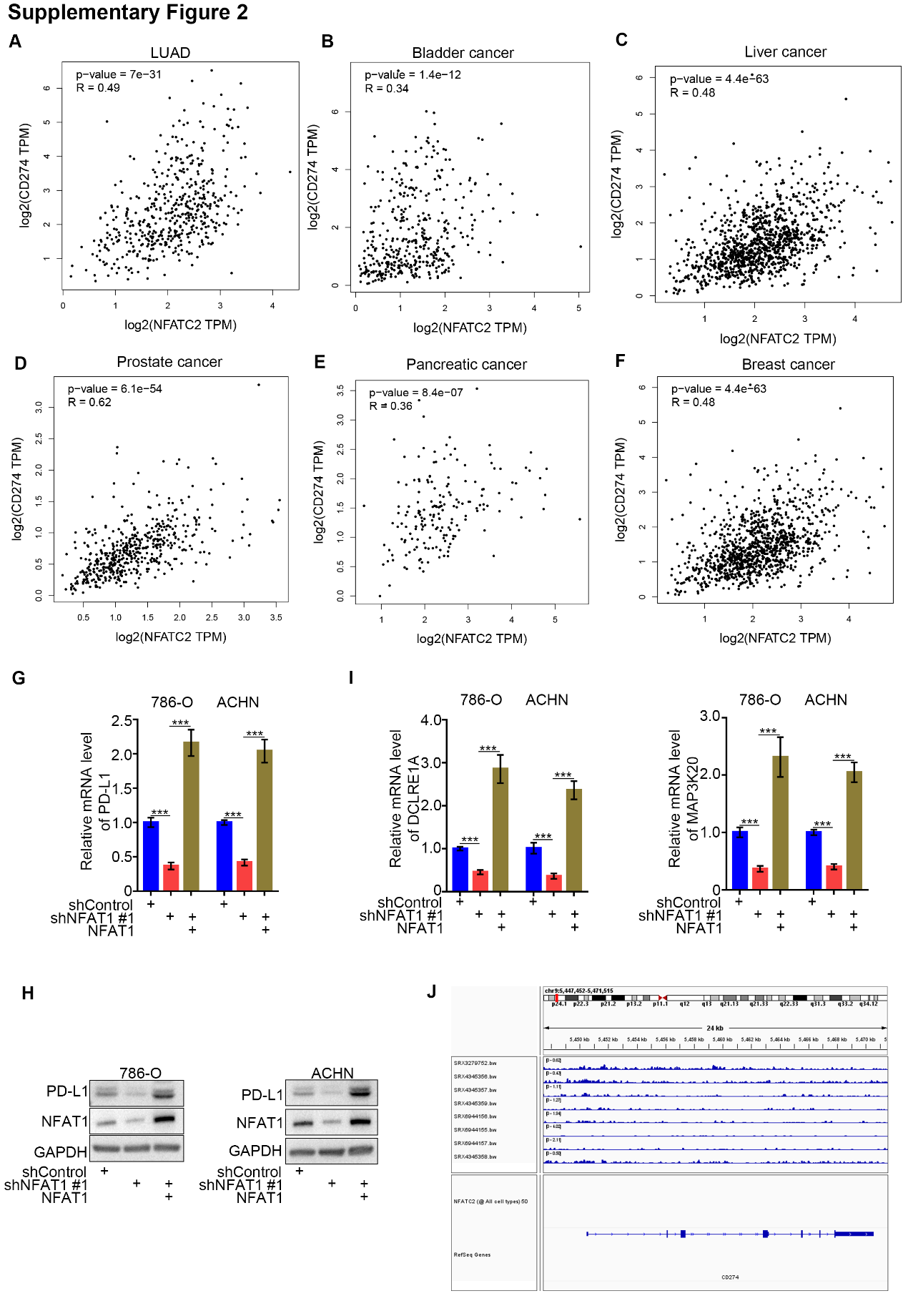


**Supplementary Figure 2. NFAT1 increased the PD-L1 expression in renal cancer cells.**

**A-F.** The GEPIA web tool was searched for the correlation between the expression of PD-L1 and NFAT1 in mRNA levels in Lung adenocarcinoma (LUAD, A), Bladder cancer (B), Liver cancer (C), Prostate cancer (D), Pancreatic cancer (E), and Breast cancer (F) samples. P values as indicated in the figure.

**G-I**. Western blot analysis and RT-PCR analysis to show the specific genes expression in RCC cells infected with sh-Control, sh-NFAT1 #1 or sh-NFAT1 #1 + NFAT1. GAPDH served as an internal reference. Data presented as the mean ± SD of three independent experiments. ***, P < 0.001.

**J.** UCSC Genome Browser was used to show there is no binding peak of NFAT1 in the promoter of PD-L1.


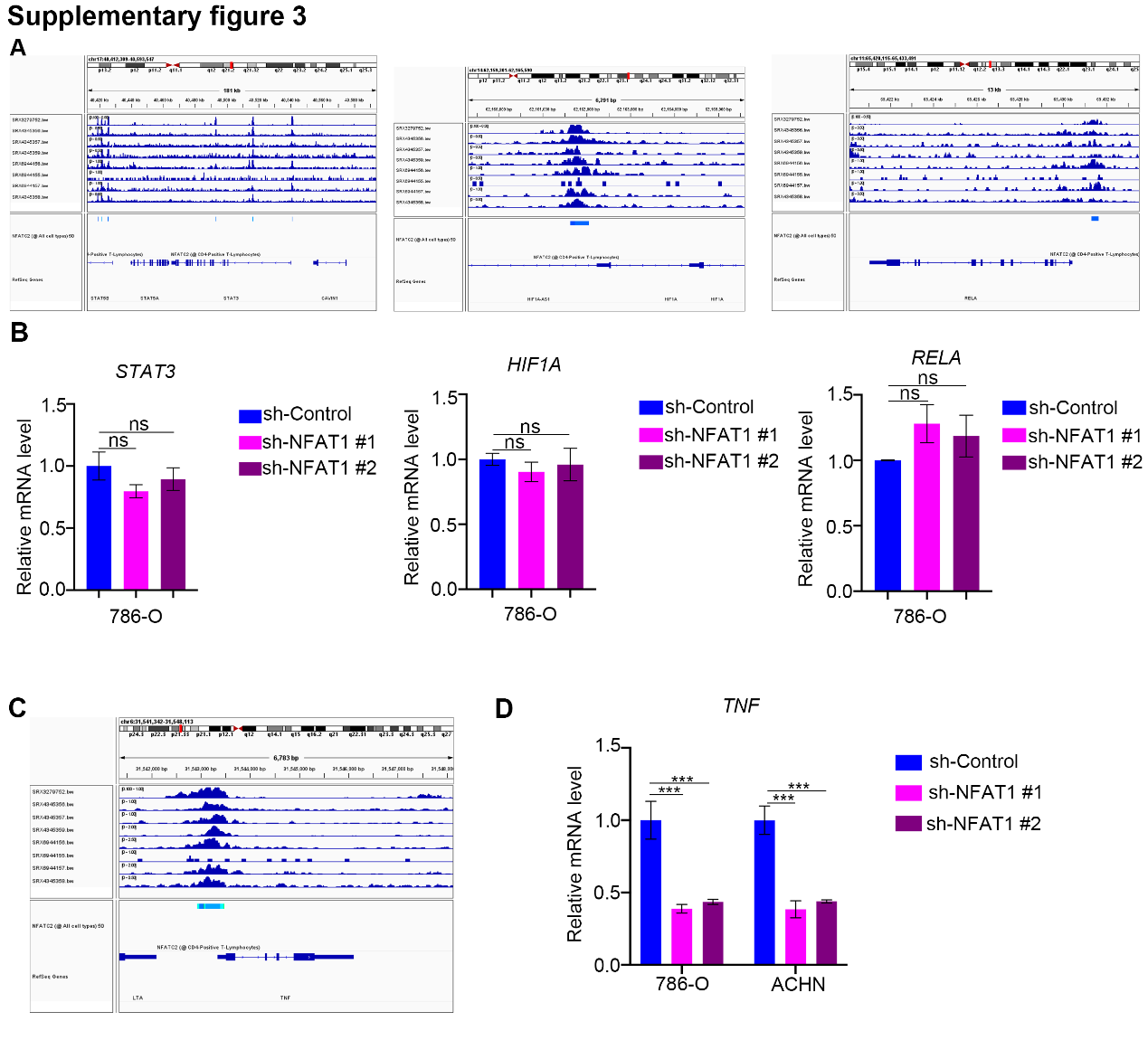


**Supplementary Figure 3. NFAT1 increased the PD-L1 expression via upregulation of TNF in renal cancer cells.**

**A**. UCSC Genome Browser was used to show there are binding peaks of NFAT1 in the promoter of *STAT3*, *HIF1A*, and *RELA*.

**B.** qRT-PCR analysis of STAT3, HIF1A, and RELA expression in renal cancer cells infected with shControl or shNFAT1 #1. GAPDH served as an internal reference. Data presented as the mean ± SD of three independent experiments. ns, not significant.

**C**. UCSC Genome Browser was used to show there is binding peak of NFAT1 in the promoter of TNF.

**D. q**RT-PCR analysis of TNF expression in renal cancer cells infected with shControl or shNFAT1 #1. GAPDH served as an internal reference. Data presented as the mean ± SD of three independent experiments. ***, P < 0.001.

**
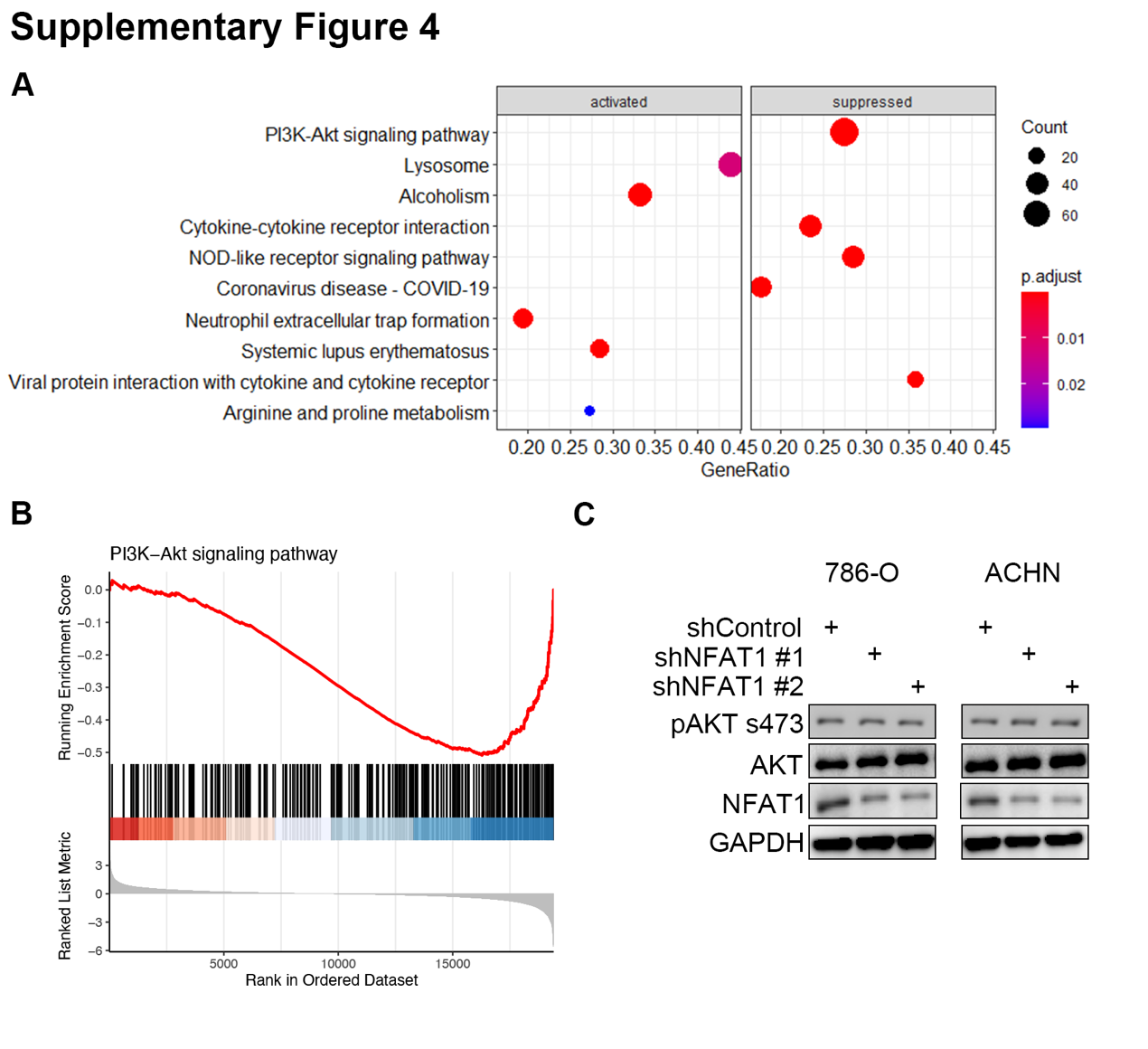
**

**Supplementary Figure 4. NFAT1 is stabilized in sunitinib resistance renal cancer cells via hyperactivation PI3K/AKT/GSK-3β signaling pathway.**

1. The KEGG enrichment analysis of RNA-seq in NFAT1 silenced 786-O cells.

**B.** Gene Set Enrichment Analysis of PI3K-AKT signaling pathway in NFAT1 silenced 786-O cells.

**C.** Western blot analysis of p-AKT expression in renal cancer cells infected with shControl or shNFAT1s. GAPDH served as an internal reference.


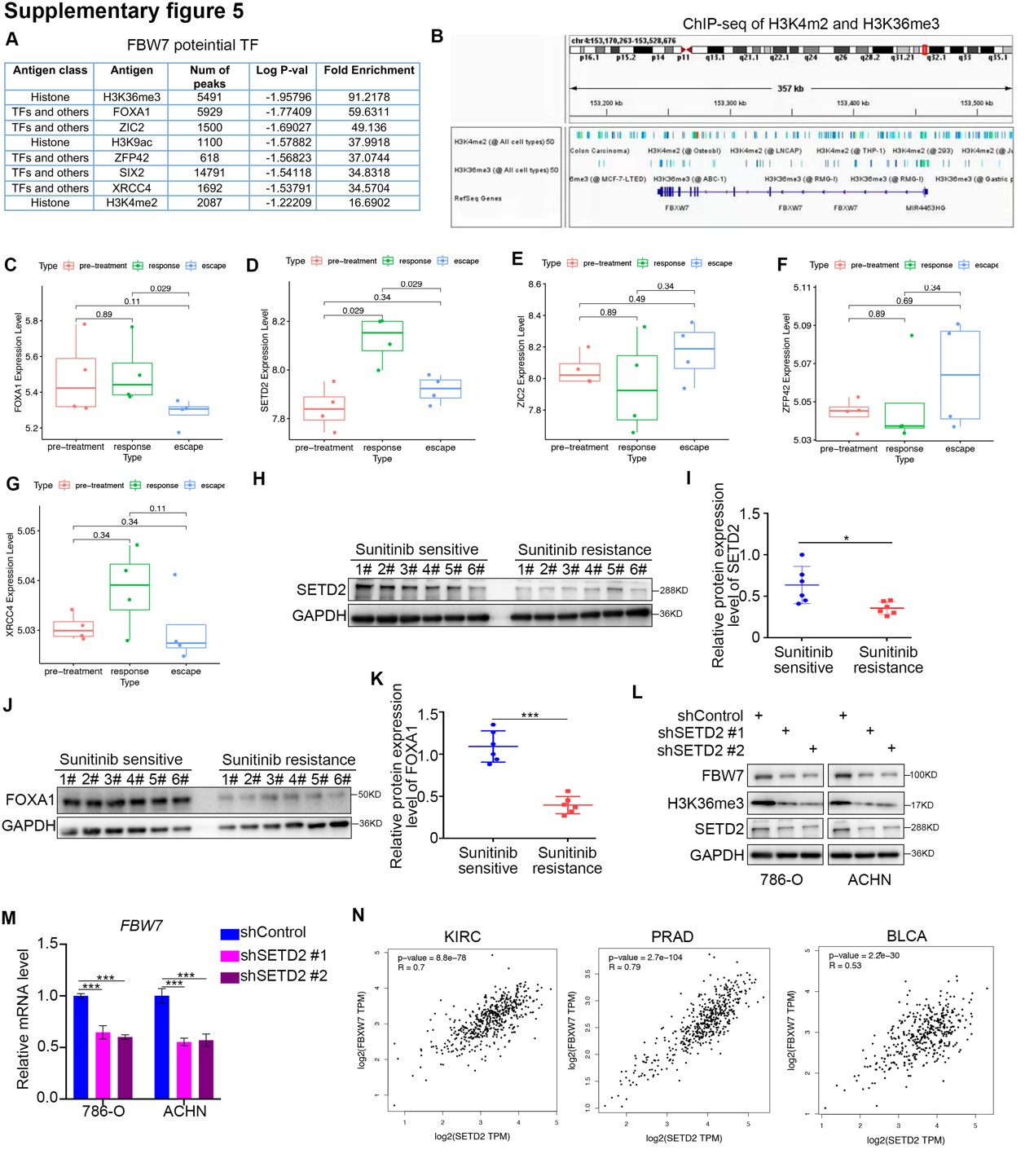


**Supplementary figure 5. FOXA1 and SETD2 induces downregulation of FBW7 expression in sunitinib-resistant RCC**

**A**. Enrichment analysis by ChIP-Atlas predicted the top 5 transcription factor and histone modification bound to the promoter region of FBW7 within ±1000bp from TSS.

**B**. UCSC Genome Browser was used to show there are binding peaks of H3K4me2 and H3K36me3 in the promoter of FBW7.

**C-G**. Relative mRNA expression level of FOXA1 (C), SETD2 (D), ZIC2(E), ZFP42(F), XRCC4 (G) in pre-treatment, response type and escape type RCC samples. The P values were shown as indicated.

**H and I.** Western blot analysis of SETD2 expression in Sunitinib sensitive and resistance RCC patients. GAPDH served as an internal reference. *, P < 0.05.

**J and K.** Western blot analysis of FOXA1 expression in Sunitinib sensitive and resistance RCC patients. GAPDH served as an internal reference. ***, P < 0.001.

**L and M.** Western blot (F) and qRT-PCR (G) analysis of FBW7 expression in renal cancer cells infected with shControl or shSETD2s. GAPDH served as an internal reference. For qRT-PCR analysis, data presented as the mean ± SD of three independent experiments. ***, P < 0.001.

**N.** The GEPIA web tool was searched for the correlation between the expression of FBW7 and SETD2 in mRNA levels in Kidney renal clear cell carcinoma (KIRC), Prostate adenocarcinoma (PRAD), and Bladder Urothelial Carcinoma (BLCA) samples. P values as indicated in the figure.

**
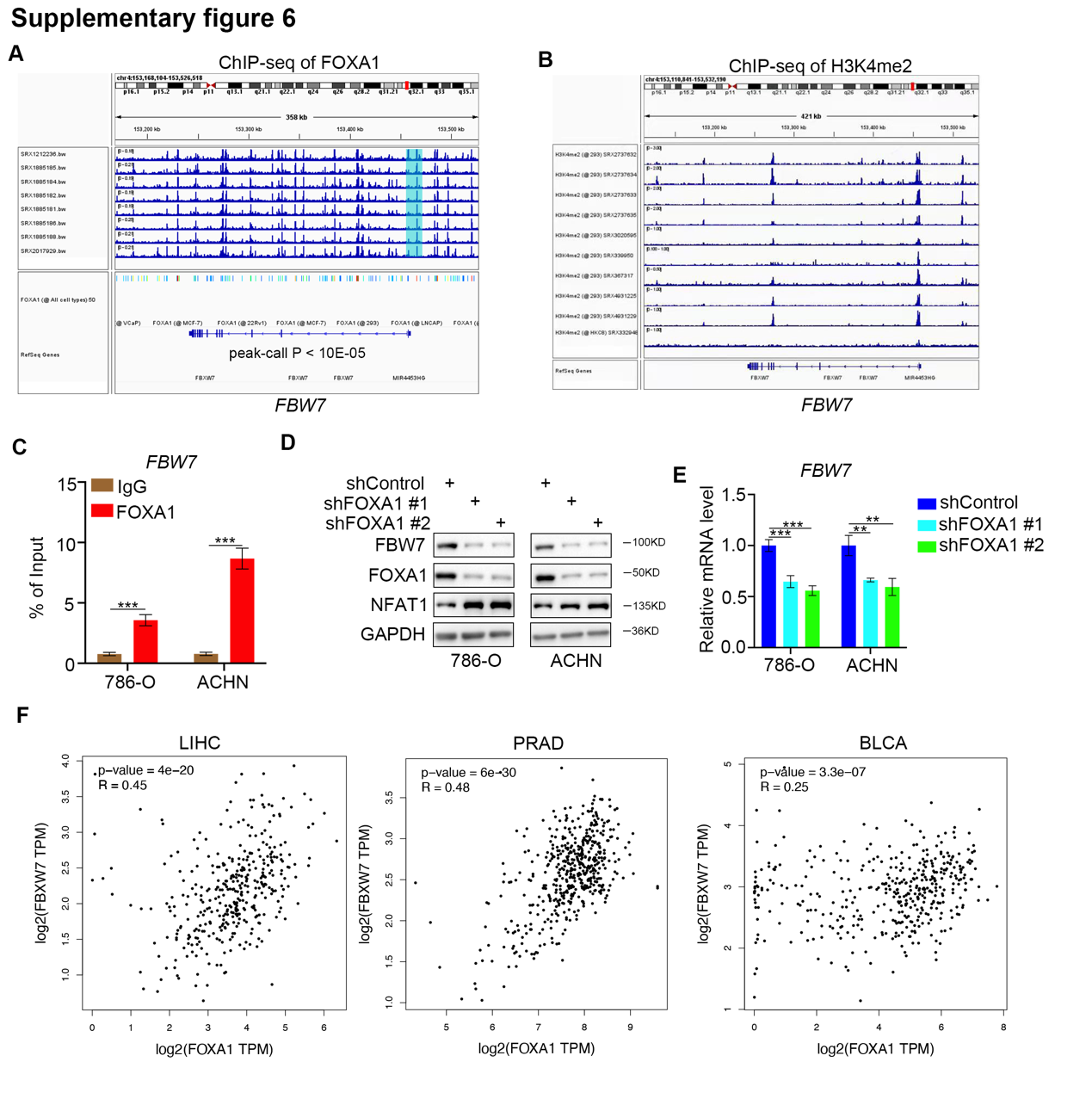
**

**Supplementary figure 6. FOXA1 and SETD2 induced the downregulation of FBW7 in sunitinib resistance RCC.**

**A and B.** UCSC Genome Browser was used to show the ChIP-seq of FOXA1 (A) and H3K4me2 (B) in the promoter of FBW7.

**C**. ChIP-qPCR of FBW7 in 786-O and ACHN cells. All data are shown as the mean values ± SD from three replicates. ns not significant; ***, P < 0.001, unpaired t test.

**D and E**. Western blot (D) and qRT-PCR (E) analysis of FBW7 expression in renal cancer cells infected with shControl or shFOXA1s. GAPDH served as an internal reference. For qRT-PCR analysis, data presented as the mean ± SD of three independent experiments. **, P < 0.01; ***, P < 0.001.

**F.** The GEPIA web tool was searched for the correlation between the expression of FBW7 and FOXA1 in mRNA levels in Liver hepatocellular carcinoma (LIHC), Prostate adenocarcinoma (PRAD), and Bladder Urothelial Carcinoma (BLCA) samples. P values as indicated in the figure

**
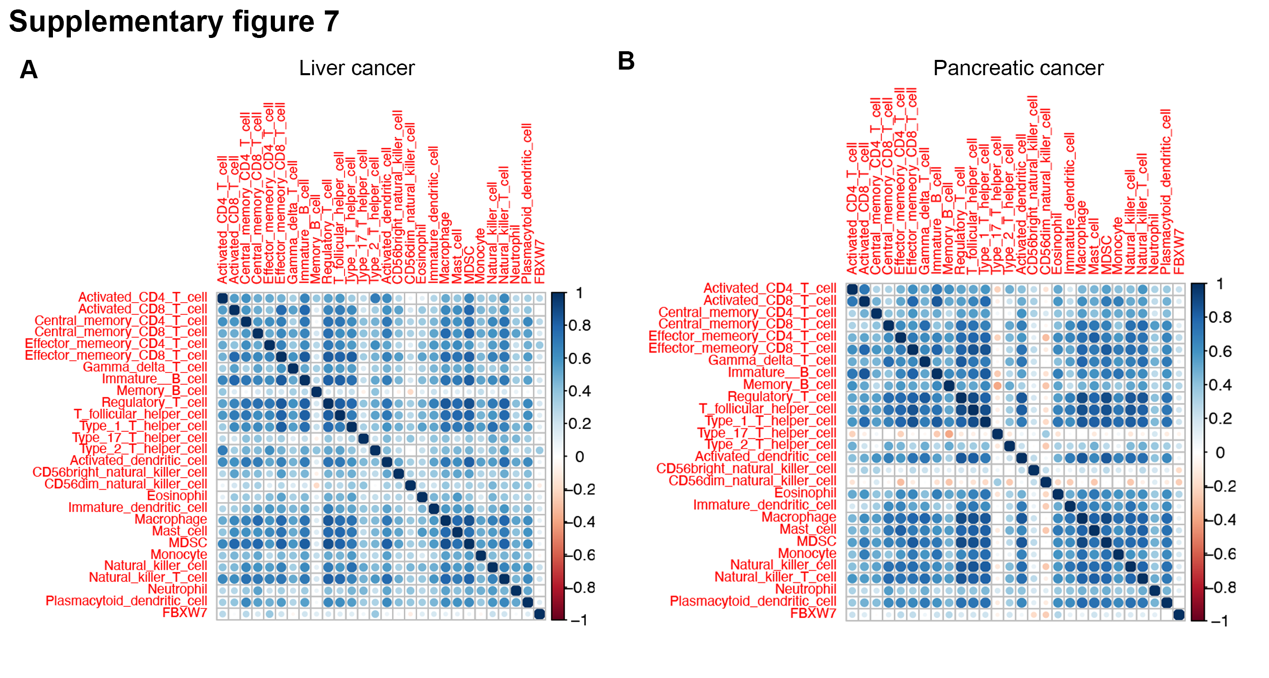
**

**Supplementary figure 7. FBW7 contributes to modulating the immune response of liver cancer and pancreatic cancer.**

**A.** The correlation between interested immune cell subsets and the expression of FBXW7 in liver cancer.

**B.** The correlation between interested immune cell subsets and the expression of FBXW7 in pancreatic cancer.

**Supplementary table 1. The primer sequences for RT-qPCR.**

| Gene | Forward primer (**5**′ - 3′) | Reverse primer (**5**′ - 3′) |
| --- | --- | --- |
| GAPDH | ATGACAATGAATACGGCTACAGCA | GCAGCGAACTTTATTGATGGTATT |
| NFAT1 | GAGCCGAATGCACATAAGGTC | CCAGAGAGACTAGCAAGGGG |
| PD-L1 | TGGCATTTGCTGAACGCATTT | TGCAGCCAGGTCTAATTGTTTT |
| FBW7 | GGCCAAAATGATTCCCAGCAA | ACTGGAGTTCGTGACACTGTTA |

**Supplementary table 2. The shRNA sequences.**

| ShNFAT1 #1 | CCGGGCTTCATTTCTGACACCTTCTCTCGAGAGAAGGTGTCAGAAATGAAGCTTTTTG |
| --- | --- |
| ShNFAT1 #2 | CCGGGCTGATGAGCGGATCCTTAAGCTCGAGCTTAAGGATCCGCTCATCAGCTTTTTG |
| ShPTEN #1 | CCGGCTAGAACTTATCAAACCCTTTCTCGAGAAAGGGTTTGATAAGTTCTAGTTTTTG |
| ShPTEN #2 | CCGGAGGCGCTATGTGTATTATTATCTCGAGATAATAATACACATAGCGCCTTTTTTG |
| ShGSK-3β #1 | CCGGGCTAGATCACTGTAACATAGTCTCGAGACTATGTTACAGTGATCTAGCTTTTTG |
| ShGSK-3β#2 | CCGGGGTATATCAAGCCAAACTTTGCTCGAGCAAAGTTTGGCTTGATATACCTTTTTG |
| ShFBW7 #1 | CCGGGCAACAACGACGCCGAATTACCTCGAGGTAATTCGGCGTCGTTGTTGC TTTTTG |
| ShFBW7 #2 | CCGGGCACTCTATGTGCTTTCATTCCTCGAGGAATGAAAGCACATAGAGTGC TTTTTG |
| ShSETD2 #1 | CCGGGCAGTAGCATCTCCACCTACTCTCGAGAGTAGGTGGAGATGCTACTGC TTTTTG |
| ShSETD2 #2 | CCGGGCAGGACACTATATCTAATAGCTCGAGCTATTAGATATAGTGTCCTGC TTTTTG |

**Table S3. The primer sequences for ChIP-qPCR.**

| Gene | Forward primer (**5**′ - 3′) | Reverse primer (**5**′ - 3′) |
| --- | --- | --- |
| FBW7 (human, FOXA1) | GCTCCCTTCCAATCCTCCCT | AAAAGCCGGACTGATCGAGG |
| TNF (human  , NFAT1) | CCTGCATCCTGTCTGGAAGT | AAAGTTGGGGACACACAAGC |
